# Polyetherureas as aqueous binders for Li ion batteries

**DOI:** 10.1039/d5gc03953c

**Published:** 2025-11-14

**Authors:** Garima Saini, Mei Jun Tan, Maximillian G. Stanzione, Ketan Pancholi, Harini Sampathkumar, Matthew Walker, Charlie Patterson, Massimo Vassalli, Aaron B. Naden, Oxana V. Magdysyuk, Jiyu Tian, A. Robert Armstrong, Amit Kumar

**Affiliations:** a EaStCHEM, School of Chemistry, University of St. Andrews North Haugh St. Andrews KY169ST UK ara@st-andrews.ac.uk ak336@st-andrews.ac.uk; b The Sir Ian Wood Building, Robert Gordon University Garthdee Rd Garthdee Aberdeen UK; c Centre for the Cellular Microenvironment, Advanced Research Centre, University of Glasgow Glasgow UK; d James Watt School of Engineering, University of Glasgow Glasgow G12 8QQ UK

## Abstract

We report here the application of polyetherureas as a new class of aqueous binder for the LiFePO_4_ positive electrode material in lithium-ion batteries. Polyetherureas have been synthesized by ruthenium-catalyzed dehydrogenative coupling of polyethylene glycol diamine and methanol avoiding conventionally used toxic diisocyanate feedstock. The best binder performance was obtained when polyetherurea was used in combination with SBR (Styrene–Butadiene Rubber), exhibiting a coulombic efficiency of ∼99.9% and a cell polarization of 30 mV. Remarkably, the combination of polyetherurea/SBR as a binder demonstrates comparable performance as that of CMC (carboxymethyl cellulose), which is a commonly used aqueous binder for lithium-ion batteries. Evidence of the involvement of polyetherureas in binder performance has been provided using IR spectroscopy and scanning electron microscopy. Physical, electrochemical, and mechanical properties of the polyetherurea have been studied using TGA, DSC, powder XRD, cyclic voltammetry, nanoindentation, tensile testing, and 180° peel test that shed light on why this polymer acts as a good binder.

## Abstract 2

This study presents polyetherureas, synthesized *via* a green, diisocyanate-free route, as a novel aqueous binder for LiFePO_4_ cathodes in lithium-ion batteries, showing comparable performance to conventional CMC binders. Image created partly using Google Gemini.

## Graphical abstract

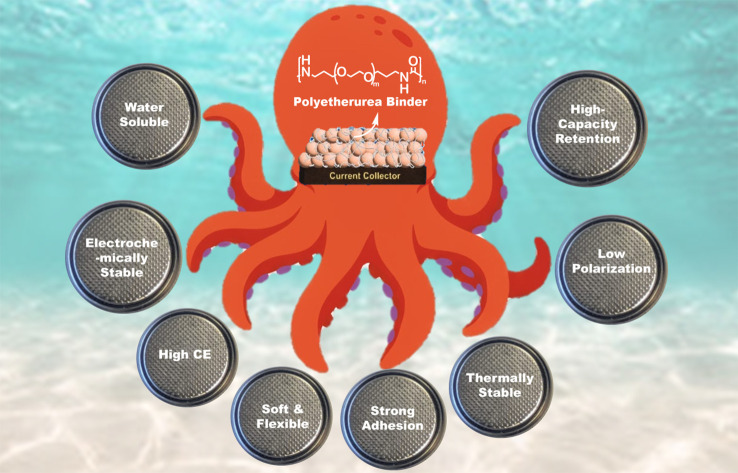

**Green foundation**

1. We present a novel water-based binder for LiFePO_4_ cathodes in lithium-ion batteries, offering a safer, more sustainable alternative to toxic, high-boiling-point solvents like NMP. With increasing regulatory restrictions on NMP, our binder addresses environmental and safety concerns while supporting the development of greener battery technologies.

2. We demonstrate a polyetherurea binder matching the performance of CMC, offering a safer, greener alternative for Li-ion batteries. Synthesized from diamines and CO_2_-derived methanol, it is semi-renewable and chemically recyclable, supporting sustainable binder development without relying on toxic isocyanates.

3. Currently, the synthesis of polyetherureas has been achieved using a ruthenium-based catalyst with a loading of 10 mol%. The synthesis can be made greener by increasing the catalytic turnover number, as well as using a catalyst of earth-abundant metal such as manganese or iron.

## Introduction

Binders are an important component of batteries that bind electrode components (*e.g.* active material, carbon additive) to each other and with the current collector.^1–4^ Although they are used in a small quantity (2–5 wt%), they play various important roles in ensuring stable electrode structure and efficient mobility of ions. The multitasking needed for binders requires them to bear several properties, such as excellent (a) adhesion, (b) thermal and electrochemical stability, (c) mechanical properties, (d) dispersion performance (with other components), (e) swelling properties (with the electrolytes), and (f) ionic conductivity.^5–7^ As a consequence, only a handful of materials have been successfully demonstrated as high-performance binders. In current-state-of-the-art lithium-ion batteries (LIBs), Carboxy Methyl Cellulose (CMC) and Styrene–Butadiene Rubber (SBR) are generally used for anodes, whereas for cathodes, non-aqueous binders such as PVDF (polyvinylidene fluoride) are used. The use of non-aqueous binders for cathodes in LIBs has a few drawbacks such as the binder needs to be dissolved in toxic and flammable solvents such as *N*-methyl pyrrolidone (NMP), causing safety concerns. Additionally, removal of NMP in the battery fabrication process takes significant energy due to its high boiling point (202 °C). Furthermore, NMP remains in the environment for extended periods and is known to emit significant amounts of greenhouse gases when burnt. Both the US Environmental Protection Agency,^8^ and European Chemical Agency^9^ have recently imposed significant restrictions on the use of NMP, which are likely to be stricter in the future. These issues have led to significant interest in the development of aqueous binders for cathode materials for lithium-ion batteries in tandem with the development of water-stable electrodes.^10–12^ In fact, several polymers such as CMC,^13^ sodium alginate,^14^ polyacrylic acid (PAA),^15^ chitosan,^16,17^ and guar gum^18^ have been evaluated as aqueous binders for cathodes in lithium-ion batteries.^19–22^ However, their performances are not much better than non-aqueous binders such as PVDF. Recently, lignin has also been reported as ultra-low dosage binder for Li–S batteries.^23^ Despite the technological advancements and development of several aqueous binders, there is no ‘one-size-fits-all’ aqueous binder, and not all the existing aqueous binders are compatible with all materials. Therefore, it is of interest to develop new aqueous binders that could allow for the effective fabrication of batteries and demonstrate high performance.

In pursuit of new aqueous high-performance binders, we hypothesized that polyureas could potentially act as an efficient binder material due to their strong mechanical and adhesion properties arising from polar functional groups.^24,25^ Additionally, polyureas could have “rigid” (derived from aromatic groups) and “flexible” (derived from aliphatic chains) segments that can provide a higher degree of elasticity, tensile strength, and adhesion, making them ideal for their use as a binder. In 2018, Sun *et al.* reported that a thin polyurea film could be utilized as an artificial SEI layer in Li-metal anodes through molecular-layer deposition.^26^ According to their hypothesis, the presence of a large number of polar groups could also redistribute the Li-ion flux, leading to uniform plating/stripping during the charging and discharging process. Considering the above-described properties of polyureas, such as adhesion, superior mechanical properties, and the recent development of polyurea to improve the SEI-layer,^26^ we hypothesized that polyureas could be used as an aqueous binder if a water-soluble polyurea could be made.

Polyureas are conventionally made from the reaction of diamines with diisocyanates, which are known to be toxic. The precursor to make diisocyanates is phosgene gas, which is even more hazardous to human health and the environment. Recent regulations have imposed stricter restrictions on the use and handling of diisocyanates, and therefore opting for a non-isocyanate route to make a battery component would be desirable for making sustainable batteries.^27^ We have recently demonstrated a new method of making polyureas from diamines and methanol feedstock, which are much safer in comparison to using isocyanate feedstock.^28,29^ Additionally, 100% renewable methanol is commercially available and can be made from the direct hydrogenation of CO_2_ making such polyureas semi-renewable.^30^ The polymerisation reaction is catalyzed by a ruthenium or manganese pincer catalyst, which dehydrogenatively couples methanol and amines to make formamides that subsequently react with amines to make urea derivatives and polyureas. Additionally, we have also demonstrated that polyureas could be hydrogenatively depolymerised back to diamines and methanol making such polymers chemically recyclable.^31^ We envisioned that diamines containing polyethyleneglycol linkages could allow us to make a water-soluble polyurea called “polyetherurea” which we hypothesized could be a potential candidate for use as an aqueous binder.

## Results and discussion

**Scheme 1:**
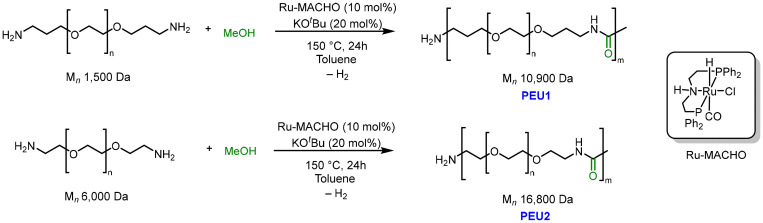
Methods used for the synthesis of polyetherureas studied in this report.

We started our investigation by developing a catalytic protocol for the synthesis of polyetherurea from the dehydrogenative coupling of polyethyleneglycol diamine and methanol. The catalytic reaction was optimized through the variation of solvent, temperature, polyethyleneglycol diamine, amount of precatalyst, and base (see SI, Table S1). The best yield of polyetherurea (∼60%) was obtained using Ru-MACHO complex (10 mol%), and KO^*t*^Bu (20 mol%) as a catalyst in toluene at 150 °C for 24 h (Scheme 1). The polyetherurea formed using this method exhibited an *M*_n_ of 10 900 Da and a polydispersity index (*Đ*) of 1.4. The polyetherurea was further characterized by NMR and IR spectroscopy as well as MALDI-TOF mass spectrometry (see SI, section 5.2). Thermal stability of PEU1 was studied by TGA (Thermogravimetric Analysis), which showed that PEU1 slowly decomposes between 220–400 °C, where *T*_d, onset_, and *T*_d, 5%_ were found to be 238 °C and 330 °C. The temperature of 90% mass loss was found to be 385 °C, which is slightly higher than that reported for CMC (295 °C).^32^ This is suggestive of the high thermal stability of the PEU1, which is an important criterion of a good binder. Additionally, the presence of higher crystallinity in a polymer such as PVDF has shown enhanced performance of the binder in the past due to the increased adhesion and viscosity.^33^ In line with this, the PXRD analysis of the PEU1 showed that the polymer is crystalline in nature with the crystallite size of ∼70 nm and the unit cell volume of 1686 Å^3^, suggestive of PEU1's ability to act as a good binder (see SI, Fig. S17).

**Fig. 1:**
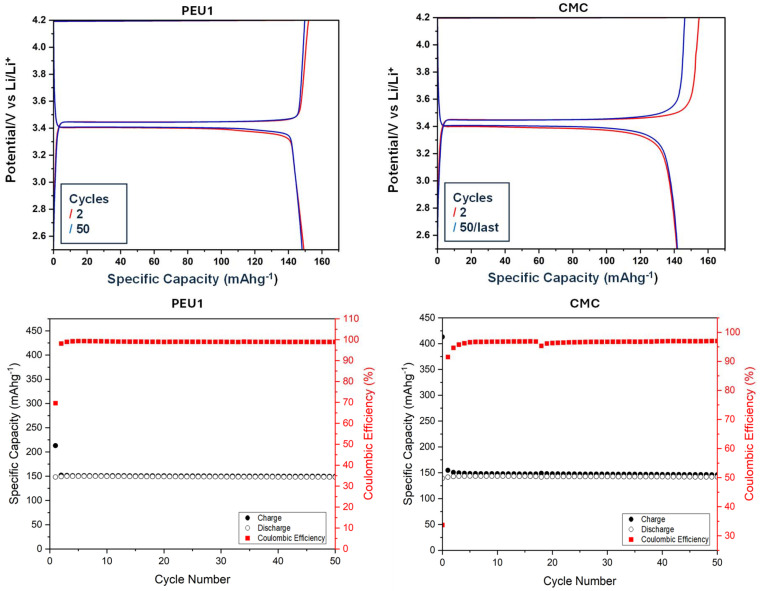
Specific capacity against potential (top), and against coulombic efficiency (bottom) for PEU1 and CMC with a 10 mA g^−1^ current density (0.06C current rate), cycled over the voltage window 2.5 V to 4.2 V.

This polyetherurea (PEU1) was tested on commercial LiFePO_4_ (LFP) by galvanostatic cycling to monitor coulombic efficiency, charge, and discharge capacity, rate performance, and differential capacity analysis (d*Q*/d*V*). Interestingly, LFP with polyetherurea (PEU1) binder exhibited a promising discharge capacity of 148 mAh g^−1^, which is close to the theoretical discharge capacity of LFP. After 50 cycles, 98.9% of the initial capacity was retained (Fig. 1). Furthermore, it showed a very high coulombic efficiency (99.2%) for 130 cycles while retaining a high specific capacity (148 mAh g^−1^), and lower polarization (0.05 V, Fig. S44–S46, SI). This retention in the discharge capacity and excellent coulombic efficiencies over the course of the cycling are desirable as they indicate no significant degradation of the battery.

We also compared the performance of PEU1 binder with that of CMC. CMC showed a maximum discharge capacity of 142 mAh g^−1^ and a capacity retention of 99.8%. In comparison, the polyetherurea (PEU1) exhibited a maximum discharge capacity of 148 mAh g^−1^ and a capacity retention of 98.9% over 130 cycles at the same rate of 10 mA g^−1^ over the voltage window 2.5–4.2 V. In addition, the polyetherurea PEU1 exhibited a higher coulombic efficiency of 99.2% compared to CMC's 95.3% indicating fewer parasitic side reactions leading to longer cycle life (Fig. 1).^34,35^ The rate capability and differential capacity analysis, (shown in the SI, Fig. S26, S27, S43 and S51) indicate similar charge and discharge capacities even at higher current rates and comparable polarization of 0.05 V for PEU1 and 0.07 V for CMC. This comparable performance is promising as it demonstrates that the polyetherurea can act as an aqueous binder equivalent to CMC. We speculate this could be because of polar functional groups (urea or alkoxy) present in the polyetherurea that could assist in the redistribution of lithium-ion flux.

**Fig. 2:**
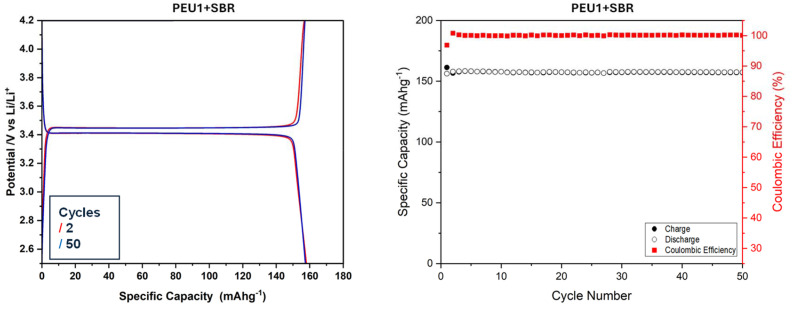
Specific capacity against potential (left), and against coulombic efficiency (right) for polyetherurea and SBR combination as a binder with a 10 mA g^−1^ current density (0.06C current rate), cycled over the voltage window 2.5 V to 4.2 V.

SBR (styrene–butadiene rubber) is commonly added to CMC to form a composite that enhances the dispersive capabilities, flexibility, and binding strength and improves the electrochemical performance of electrodes.^7,36–38^ We, therefore, studied the combination of polyetherurea (PEU1) with SBR (50 : 50) as an aqueous binder and compared its electrochemical performance against PEU1 for LFP. Comparatively, the PEU1 + SBR has slightly higher charge and discharge capacities with stable discharge capacity throughout the cycles at 157 mAh g^−1^ (Fig. 2) and appreciable coulombic efficiency of 99.9%. In both samples (PEU1 and PEU1 + SBR), there was limited polarization, which once again exhibits that the polyetherurea is a compatible binder with LFP. Furthermore, the d*Q*/d*V* plot showed that in the case of pure polyetherurea (PEU1), the oxidation/reduction peaks occur at 3.44/3.39 V, which has a difference of 50 mV. Comparatively, in the case of polyetherurea/SBR composite, the oxidation/reduction peaks occur at 3.44/3.41 V, indicating an even smaller polarization of 30 mV (see SI, Fig. S55). These studies show that polyetherurea in combination with SBR performs as a better binder than pure polyetherurea and is comparable to that of CMC. We also tested the electrochemical performances of PEU1 combined with SBR in ratios 30 : 70 and 70 : 30. In both cases, the specific discharge capacity and coulombic efficiency were found to be less than those found with PEU1/SBR in ratio 50 : 50 (see SI, Fig. S60–S63). We then compared PEU1/SBR with CMC/SBR and found that the cells with CMC/SBR binder exhibited a specific discharge capacity of 153 mAh g^−1^ with a coulombic efficiency of about 98.0% which is slightly lower than that of PEU1/SBR (see SI, section 7.2). The cells were also run for 500 cycles at 3C current rate, and we observed promising results for both PEU1 and PEU1/SBR (see SI, Fig. S47, S48, S56 and S57).

Encouraged by the performance of PEU1, we studied the effect of the nature of the polyetherurea on the specific capacity and coulombic efficiency. We compared the galvanostatic profile of PEU1 which is made from polyethyleneglycol bispropyl diamine of *M*_n_ = 1500 Da with the polyetherurea made from polyethyleneglycol diamine of *M*_n_ = 6000 Da (PEU2 in Scheme 1) under identical catalytic conditions (see SI for the properties and characterization details of both the polyetherureas). The cycling data obtained from both polyetherureas exhibited the characteristic plateaus in both charge and discharge curves, which indicate some difference in performance, perhaps due to the long chain ethers present in the polymer (see SI, section 7.7).

To probe if the urea functional group is important for binder applications, we compared PEU1/SBR with polyethylenglycol diamine/SBR which was used as a starting material to make polyetherurea. From the d*Q*/d*V* plots (see SI), the starting material (polyethyleneglycol diamine) showed comparable capacity and pronounced polarisation compared to that of the polyetherurea (PEU1). In the starting material (polyethylenglycol diamine/SBR), the oxidation/reduction peaks occur at 3.46/3.39 V, indicating a much larger polarization of 0.07 V than that of the PEU1/SBR, which has a polarization of 0.03 V. The coulombic efficiency of polyethyleneglycol diamine/SBR (98.2%) is less than that of PEU1/SBR (99.9%). This is likely because of the increased electrode resistance when the binder has inadequate adhesion to the electrode materials, possibly due to the lower degree of hydrogen bonding present in amine in comparison to urea. In the case of polyethyleneglycol diamine/SBR, there is pronounced sloping behavior in both the charging and discharging curves which is indicative of less well-defined phase transitions in the starting material, potentially due to parasitic side reactions or increased resistance arising from poor electrode structure in later cycles. These studies suggest that the urea functionality is a positive contributor to the electrochemical performance.

**Fig. 3:**
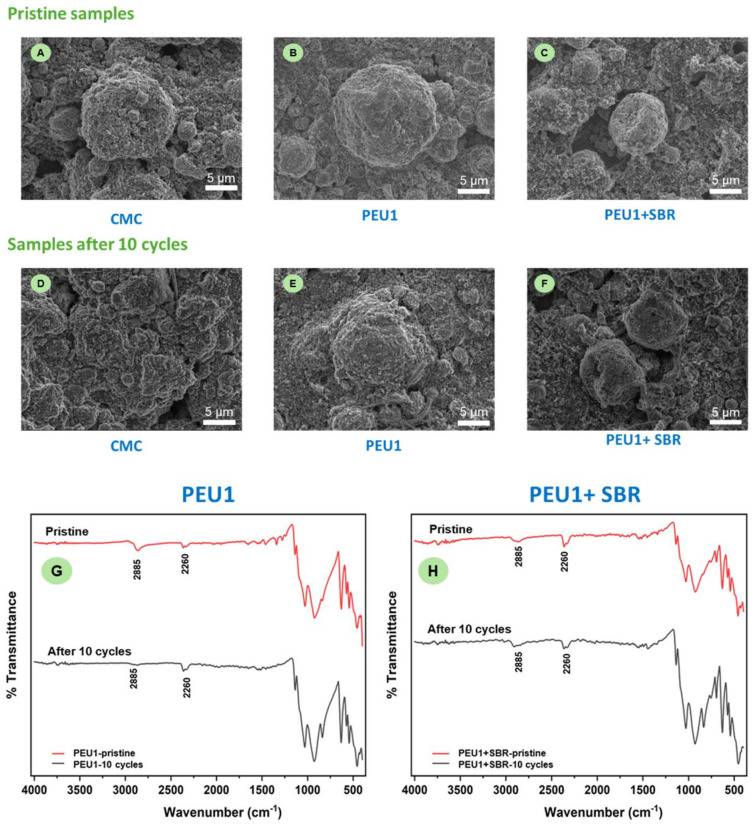
SEM images of cathode before and after galvanostatic cycling for CMC (A and D), PEU1 (B and E) and PEU1 + SBR (C and F) at 10 mA g^−1^ current density (0.06C current rate). IR spectra of cathode before and after galvanostatic cycling for PEU1 (G) and PEU1 + SBR (H) at 10 mA g^−1^ current density (0.06C current rate).

To investigate if the polyetherureas indeed are acting as binders, we analyzed the electrode after 10 cycles using Scanning Electron Microscopy and IR spectroscopy. As seen in Fig. 3, the morphology of the surface before and after galvanostatic cycling looks similar in the case of cells where PEU1 and PEU1 + SBR were used as binders. No obvious cracking is observed suggesting that the polymer is acting as a good binder to keep the electrode intact. In contrast, morphology appears to be changed in the case of the CMC binder. This supports our results of polyetherurea being a promising binder for LFP cathode material in lithium-ion batteries (Fig. 3). Additionally, the IR spectrum before and after galvanostatic cycling appears the same qualitatively when PEU1 + SBR was used as a binder. However, some signals *e.g.* at 2885 cm^−1^ that would correspond to the N–H stretch of the polymer appear to diminish when just PEU1 was used as a binder whereas this is not the case for PEU1 + SBR (Fig. 3). This further confirms that polyetherurea + SBR is a more promising binder than just polyetherurea for lithium-ion batteries.

**Fig. 4:**
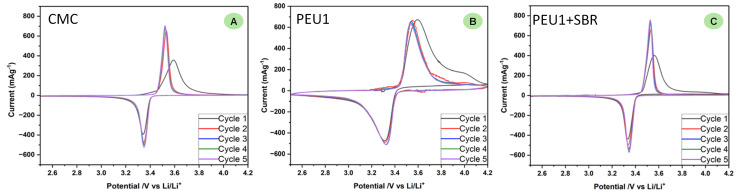
Cyclic voltammograms showing 5 cycles of the half cells cycled at 0.1 mV s^−1^ containing different binders CMC (A), PEU1 (B) and PEU1 + SBR (C).

We carried out further studies to understand the properties of polyetherurea (PEU1) that make it a good binder. One of the important properties of a polymeric binder is its electrochemical inertness. This was probed by conducting cyclic voltammetry studies of half cells made using PEU1, PEU1 + SBR, and CMC binders between a voltage window of 2.5 V to 4.2 V (Fig. 4). In all these cases reversible redox processes were observed suggestive of the electrochemical stability of these materials. Additionally, a slightly higher polarisation was observed in case of PEU1 (reduction at 3.32 V and oxidation at 3.52 V, and Δ*V* = 200 mV) in comparison to that of PEU1 + SBR (reduction at 3.34 V and oxidation at 3.52 V, and Δ*V* = 180 mV). Furthermore, the relatively sharper shape in the case of PEU1 + SBR is also suggestive of a single step lithiation/delithiation process whereas this could be a more gradual process in the case when just PEU1 was used as a binder. These data suggest that PEU1 + SBR is a better-performing binder than PEU1 as also discussed above. The redox behaviour in the case of PEU1 + SBR was found to be similar to that of CMC binder with sharp shapes and polarisation of 170 mV (reduction at 3.35 V and oxidation at 3.52 V).

**Fig. 5:**
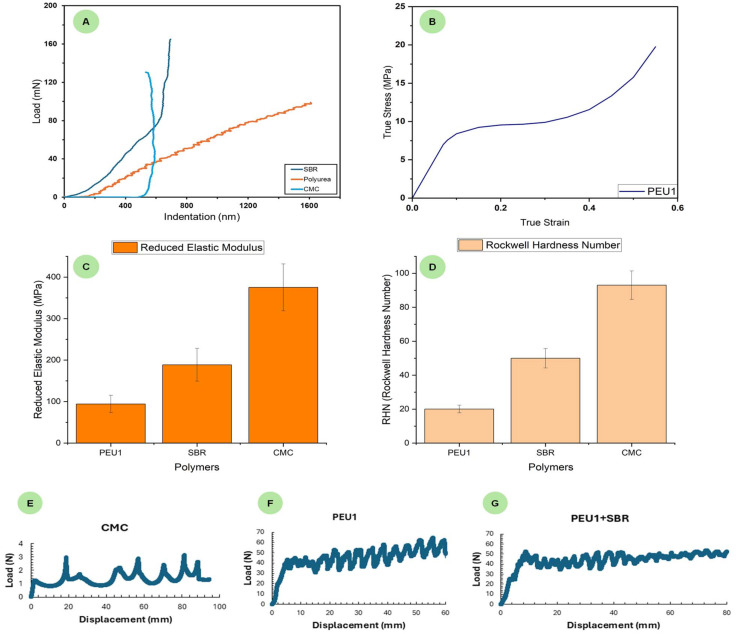
Load *vs.* indentation curve from nanoindentation studies (A), and stress *vs.* strain curve for PEU1 (B). values of reduced elastic modulus (C) and Rockwell hardness numbers (D) for CMC, SBR, and PEU1. Load *vs.* displacement plots from 180° peel test of electrodes with binders CMC (E), PEU1 (F) and PEU1 + SBR (G).

To get insights into the mechanical properties of binders, films (2 mm thickness) of PEU1, SBR, and CMC were prepared and studied using the nanoindentation technique (Fig. 5A). As shown in Fig. 5C, the reduced elastic modulus of PEU1 (95 MPa) was found to be lower than those of SBR (189 MPa) and CMC (375 MPa). This could suggest that PEU1 is relatively more flexible and can change its shape and size considerably. This is beneficial for a binder as a flexible material can help prevent cracking and particle detachment during cycling. Similarly, the Rockwell hardness number of PEU1 (20) was found to be lower than those of SBR (50), and CMC (93) suggesting that PEU1 is a softer material in comparison to SBR and CMC (Fig. 5D). Performing the tensile testing on the PEU1 sample (stress/strain study, Fig. 5B, see SI for details) showed the yield strength to be 7.6 MPa which is lower than that of CMC (∼18 MPa) and similar to that of PVDF (<10 MPa).^39^ This would suggest that in terms of elasticity, PEU1 is similar to PVDF but inferior to CMC. The stress at failure (tensile strength) was measured to be (∼18 MPa) for PEU1.

Furthermore, the adhesive properties of these binders were studied by a 180° peel test (Fig. 5E–G). The adhesion force or CMC was found to be 0.7 N cm^−1^ which is only slightly lower than a reported value in the past (1.1 N cm^−1^).^40^ However, remarkably, the adhesive force in the case of PEU1 calculated using the same methodology was found to be 19.24 N cm^−1^ whereas the adhesive force for PEU1 + SBR was found to be 18.5 N cm^−1^. These data suggest that PEU1 has a much stronger adhesive property than CMC making it a desirable candidate for a high-performance binder. Polyureas are known to act as very strong adhesives and therefore these results are consistent with that.^41,42^

## Conclusion

In conclusion, we have demonstrated polyetherurea to be a new class of aqueous binder for lithium-ion batteries. The best results are obtained when polyetherurea is used in combination with SBR leading to a small polarization of 30 mV and exhibiting a higher specific capacity of 157 mAh g^−1^ and a coulombic efficiency approaching 100% for LFP. Such attributes are comparable with the CMC binder, which is a commonly used aqueous binder for lithium-ion batteries. Further studies of the electrode material using SEM and IR spectroscopy suggest that indeed the polyetherurea (PEU1) keeps the cell intact acting as a suitable binder. Studying various properties of PEU1 revealed that PEU1 is thermally very stable (*T*_d,90%_ = 385 °C), crystalline in nature, electrochemically stable (between 2.5 V to 4.2 V), and softer than CMC and SBR while exhibiting a moderate yield strength (7.6 MPa). Most remarkably, the PEU1 has significantly higher adhesion force in comparison to CMC or SBR. These properties clearly support the ability of PEU1 to act as a promising binder for lithium-ion batteries. A comparative summary of various properties and performance of PEU1 or PEU1 + SBR with respect to CMC or CMC + SBR binder has been provided in SI, Table S2. Thus, we believe that the proof of concept reported here opens up new avenues to use polyetherureas as aqueous binders with various electrode materials in different types of alkali metal batteries.

## Conflicts of interest

There are no conflicts to declare.

## Supplementary Material

GC-028-D5GC03953C-s001

## Data Availability

The raw research data supporting this publication can be accessed at https://doi.org/10.17630/18007bfa-ba92-4cd2-86af-ce504e640f91 Supplementary information (SI): data related to the synthesis and characterisation of polymers as well as studies related to binder tests. See DOI: https://doi.org/10.1039/d5gc03953c.
