# Supplementary material for: Polyetherureas as aqueous binders for Li ion batteries
Source: Green Chem. 2025 Nov 14;28(1):318–25. doi: 10.1039/d5gc03953c (PMC12645821; doi:10.1039/d5gc03953c)
Supplement: GC-028-D5GC03953C-s001 [file GC-028-D5GC03953C-s001.pdf]

Supplementary Information (SI) for Green Chemistry.
This journal is © The Royal Society of Chemistry 2025

Electronic Supporting Information for Manuscript:

Polyetherureas as aqueous binders for Li ion Batteries

Garima Saini,[a]† Mei Jun Tan,[a]† Maximillian G. Stanzione,[a] Ketan Pancholi,[b] Harini Sampathkumar,[b] Matthew Walker,[c][d] Charlie Patterson,[d] Massimo Vassalli,[c][d] Aaron B. Naden,[a] Oxana V. Magdysyuk,[a] Jiyu Tian,[a] A. Robert Armstrong,*[a] Amit Kumar*[a]

[a]EaStCHEM, School of Chemistry, University of St. Andrews, North Haugh, St. Andrews, KY169ST, UK.

[*] A. Robert Armstrong: ara@st-andrews.ac.uk

[*] Amit Kumar: ak336@st-andrews.ac.uk

[†] MJT and GS contributed equally.

[b]The Sir Ian Wood Building, Robert Gordon University, Garthdee Rd, Garthdee, Aberdeen, UK.

[c]Centre for the Cellular Microenvironment, Advanced Research Centre, University of Glasgow, Glasgow, UK.

[d]James Watt School of Engineering, University of Glasgow, Glasgow G12 8QQ, U.K.

Table of Contents

Experimental Details 3
1. General Considerations 3
2. Procedure for the synthesis of polyetherurea 4
General method for the synthesis of polyetherurea under closed conditions: 4
3. Table S1: Optimisation of Catalytic conditions 5
4. Method to calculate the NMR yield of polyurea 5
5. Characterisation 6
5.1. PEG diamine, Mn=1500 6
5.2. Characterisation Details for the Isolated Polyurea PEU1 9
5.3. Characterisation Details for the Isolated Polyurea PEU2 16
6. Procedure for making coin cells 20
7. Galvanostatic Data 21
7.1. CMC 21
7.2. CMC with SBR 23
7.3. PEG diamine, Mn=1500 25
7.4. PEG Diamine, Mn=1500 with SBR 26
7.5. PEU1 28
7.6. PEU1 with SBR 34
7.7. PEU2 40
8. SEM images of electrodes before and after cycling 41
9. ATR-FTIR images of electrodes before and after cycling 43
10. Impedance data 43
11. Mechanical properties of binders 43
12. Table S2: Comparison of properties/performance of PEU1/PEU1+SBR binder against those of CMC binder. 45
13. Table S3: Comparison of our results with the other aqueous binders reported in the literature 46
14. Atom economy 46
15. References 47

Experimental Details

1. General Considerations

All manipulations, unless otherwise stated, were performed under an argon atmosphere using standard Schlenk line and glove-box techniques. Glassware was oven-dried at 130 °C overnight and flamed under vacuum prior to use. THF and Toluene were dried using a Grubbs-type solvent purification system (Innovative Technologies SPS) equipped with a degasser. PEG Diamine, Mn= 1500, PEG Diamine, Mn= 6000 and ruthenium complex **Ru-MACHO** were purchased from Sigma-Aldrich and TCI and used as received.

KO^tBu was purchased from Sigma-Aldrich and stored at 80 °C and dried before use. NMR solvents were purchased from Sigma-Aldrich and used as received.

The LFP ($LiFePO_4$) was supplied by AMTE Power (now Lionvolt) and used as received. The conductive carbon was purchased from Imerys and dried before use.

NMR spectra were recorded on a Bruker AVIII-HD 500 MHz NMR spectrometer at 298 K unless otherwise specified. Residual proton solvent was used as reference for ^1H spectra in deuterated solvent samples. All chemical shifts (δ) are quoted in ppm and coupling constants (*J*) in Hz.

Gel permeation chromatography (GPC) was performed on an Agilent 1260 InfinityLab II GPC fitted with a refractive index (RI) detector (35 °C). The single (plus guard column) Agilent PolarGel column setup was contained within an oven (35 °C). H_2O was used as the eluent at a flow rate of 1.0 mL min^{-1}. Samples were dissolved in the eluent (2.0 mg mL^{-1}), filtered (0.2 µm pore size) and run immediately. The calibration was conducted using a series of monodisperse poly(ethylene glycol) (M_n = 194–20,000 g mol^{-1}) and poly (ethylene oxide) (M_n = 30,000–50,000 g mol^{-1}) standards obtained from Agilent Technologies.

Infrared spectra (ATR-FTIR) were collected using a Shimadzu IRAffinity-1. TGA was performed using a Stanton Redcroft STA-780 Series Thermal Analyser between 20–700°C at a heating rate of 10 °C/min under a flow of nitrogen gas (25 mL/min). DSC analyses were performed using a Netzsch DSC204 between –80–300°C at a heating rate of 10 °C/min under a flow of nitrogen gas (20 mL/min) after an initial heat/cool cycle (25–120 °C at 10 °C/min with a 20-minute isothermal at 120 °C) to remove the thermal history of the sample. A JEOL JSM-IT800 scanning electron microscope (SEM) was used to record the microstructures and EDS elemental analysis of the pristine electrodes and electrodes after running for 10 cycles.

Cyclic voltammetry (CV) and Galvanostatic cycling were performed on a Biologic BCS-805 cycler and Neware BTS4000-5V10mA battery testing system, both at 30°C. CV was conducted in a cell at

0.1 mV s^{-1} for 5 cycles from 4.2 to 2.5 V. The galvanostatic charge and discharge were performed with a potential range between 2.5 V and 4.2 V. The cycling performance was measured at different current densities of 10, 25 and 100 mAg^{-1} for 50-200 cycles. The rate capabilities of the half-cells were tested at 25, 50, 75, 100, 250 and 500 mAg^{-1}. The coin cells were prepared using LFP:Carbon:Binder in the ratio of 80:10:10 as cathode with a Al foil as the current collector, Li-metal (1 cm^2 area, thickness 0.1 cm) as the counter electrode, 1M LiPF6 in ethylene carbonate: dimethyl carbonate (1:1) as electrolyte. The theoretical capacity of 170 mAhg-1 is used for LFP to calculate C-rates for electrochemical testing.

2. Procedure for the synthesis of polyetherurea

General method for the synthesis of polyetherurea under closed conditions:

PEU1

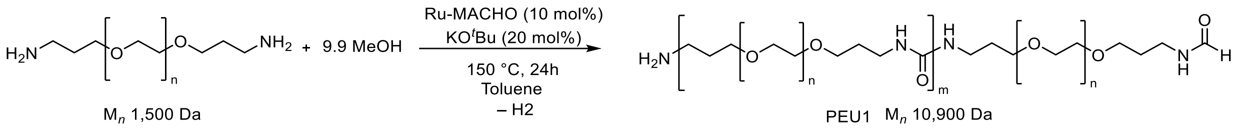

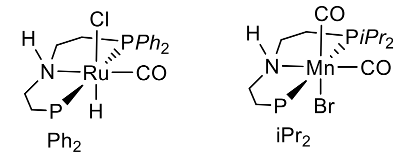

Ru-MACHO **Mn-MACHO**

This experiment was performed using standard Schlenk line techniques.

PEG-diamine, Mn=1500 (0.25 mmol, 1 eq., 375 mg), Ru-MACHO complex (10 mol%, 0.1 eq., 15.1758 mg), potassium tert-butoxide (20 mol%, 0.2 eq., 5.6106 mg), toluene (0.5 mL) and methanol (0.1 mL, 9.9 eq.) were added to a J-Young Flask and sealed under Argon. The mixture was then heated to 150℃ and stirred at 400 rpm for 24 hours. After this time, the reaction flask was allowed to cool to room temperature. Hexane (approx. 20 mL) was added to aid precipitation, and the reaction mixture was cooled to 5°C for 24 hours to afford a yellow solid. The crude product was collected by filtration, washed 3 times with hexane and Diethyl ether, and dried under reduced pressure to afford the desired polyetherurea (PEU1, 230.4 mg, 0.15 mmol, 60 %).

PEU2

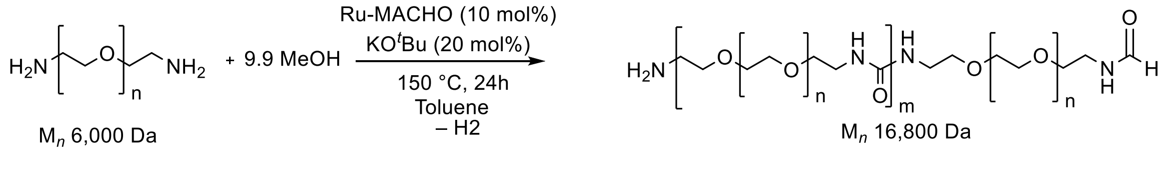

This experiment was performed using standard Schlenk line techniques.

PEG-diamine, Mn= 6000 (0.0167 mmol, 1 eq., 100.2 mg), Ru-MACHO (10 mol%, 0.1 eq., 1.0137 mg), potassium tert-butoxide (20 mol%, 0.2 eq., 0.3748 mg), toluene (0.5 mL) and methanol (0.1 mL, 9.9 eq.) were added to a J-Young Flask and sealed under Argon. The mixture was then heated to 150℃ and stirred at 400 rpm for 24 hours. After this time, the reaction flask was allowed to cool to room temperature. Hexane (approx. 20 mL) was added to aid precipitation, and the reaction mixture was cooled to 5°C for 24 hours to afford a yellow solid. The crude product was collected by filtration, washed 3 times with hexane and Diethyl ether, and dried under reduced pressure to afford the desired polyetherurea (PEU2, 89.6 mg, 0.0149 mol, 89%).

3. Table S1: Optimisation of Catalytic conditions

Entry No.	Duration (h)	Temperature (°C)	Solvent	Solvent (mL)	Starting material[a] (M_n), Da	Starting material (mmol)	Catalyst (mol%)	KOtBu (mol%)	Methanol (mL)	NMR yield of Polymer (%)	Polymer molar mass (Mn), Da	Đ	TON	TOF (h^{-1})
1	48	130	THF	2	1500	0.250	2.5	5.0	0.4	0	-	-	0	0
2	24	130	Toluene	2	1500	0.250	2.5	5.0	0.4	12	6600	1.1	4.8	0.2
3	24	150	Toluene	0.50	1500	0.250	2.5	20.0	0.1	26	12,100	1.2	10.4	0.4
4	24	150	Toluene	0.50	1500	0.250	10.0	20.0	0.1	60[b]	10,900	1.4	6	0.25
5	24	150	Toluene	0.50	1500	0.250	20.0	40.0	0.1	25	12,000	1.2	2.5	0.
6	24	150	Toluene	0.50	230	0.412	10.0	20.0	0.1	0	-	-	0	0
7	24	150	Toluene	0.50	6000	0.017	10.0	20.0	0.1	89[b]	16,800	2.0	8.9	0.4
8[c]	24	150	Toluene	0.50	1500	0.250	10.0	20.0	0.1	32	6,763	1.2	3.2	0.13

[b] isolated yields. [c] Mn-MACHO is used as pre-catalyst.

[a]Starting materials:

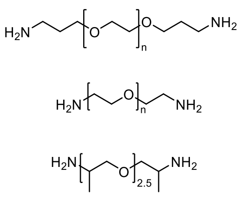

M_n ~1500

M_n ~6000

M_n ~230

4. Method to calculate the NMR yield of polyurea

At the end of the reaction, 31 mg of the product, an internal standard (0.2 mmol of 1,1-diphenylethylene) was added to DMSO-d6 (0.5 mL), and the sample was analysed by ^{1}H NMR spectroscopy. As shown in the following figure, the signal at δ6 ppm was assigned to urea protons

(*NH*), and the signal at δ5.5 ppm was assigned to 1,1-diphenylethylene (C*H₂*). The yield of polyurea was estimated by the integration of these two signals.

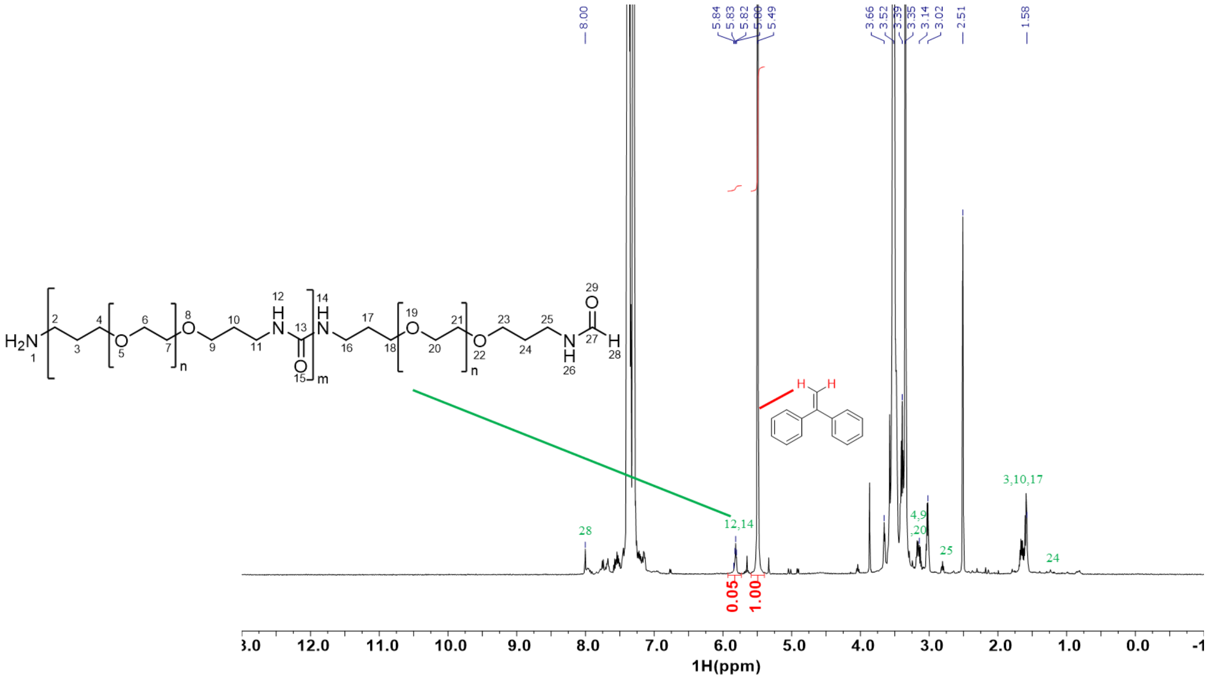

Figure S1. ^{1}H NMR (500 MHz, DMSO-d_6) spectrum of PEU1 highlighting the urea protons at 6.0 ppm and the protons of 1,1-diphenylethylene at 5.5 ppm.

5. Characterisation

5.1. PEG diamine, Mn=1500

^{1}H NMR (500 MHz, DMSO-d_6): δH 3.59 (m, H-3,4,5,6), 2.67 (m, H-1,8), 1.67-1.66 (m, H-2,7)

^{13}C{^{1}H} NMR (126 MHz, DMSO-d_6): δC 69.53(C-4,5), 37.64(C-1,8)

IR (ATR-FTIR, cm^{-1}): ν 2875 (N-H, stretch), 1465 (N-H, bend), 1340,1278 (C-N, stretch),1101(C-O, stretch), .

GPC: Mn = 1500

TGA Profile: T_{donset}= 169°C, $T_{d,\ 5\%=}$ 305°C

DSC trace*:* Tg= -6.0 °C, Tm= 47.8 °C, Tc= 14.2°C

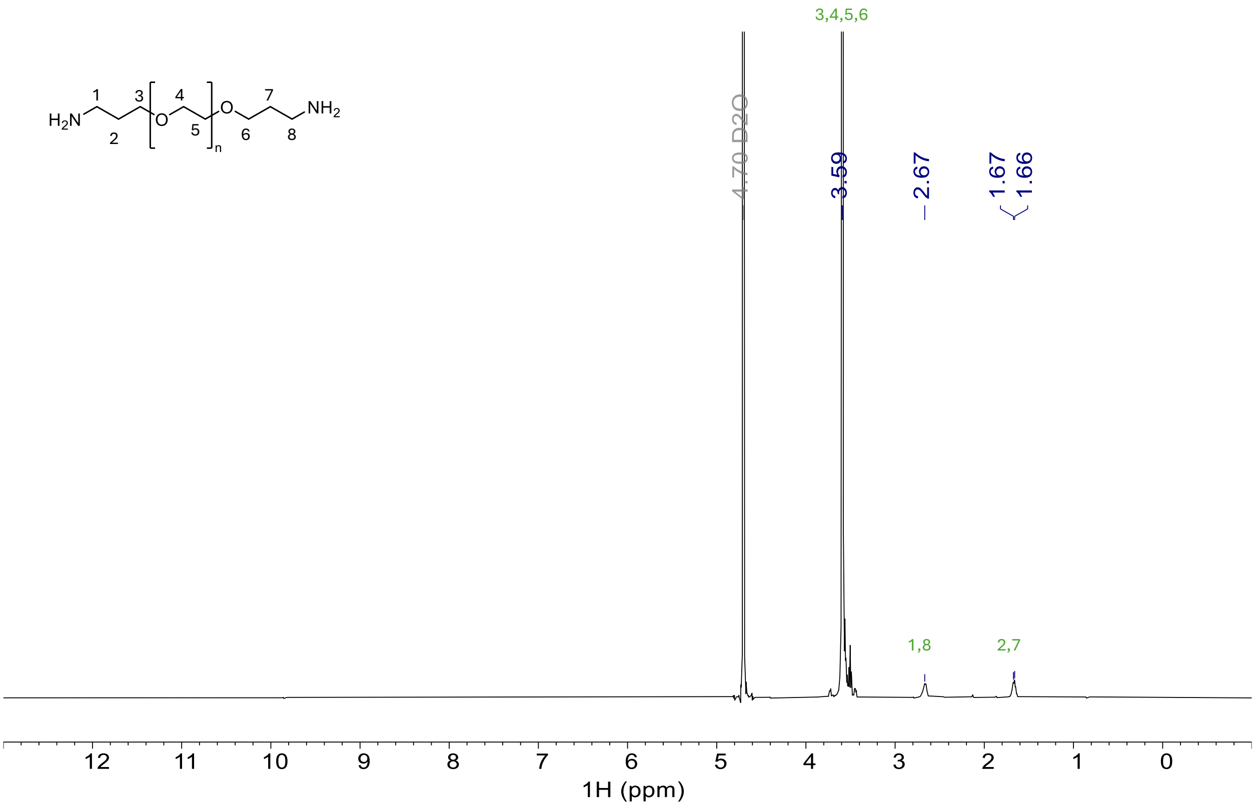

Figure S2. ^{1}H NMR (500 MHz, D_2O) spectrum of the starting material PEG diamine, Mn=1500.

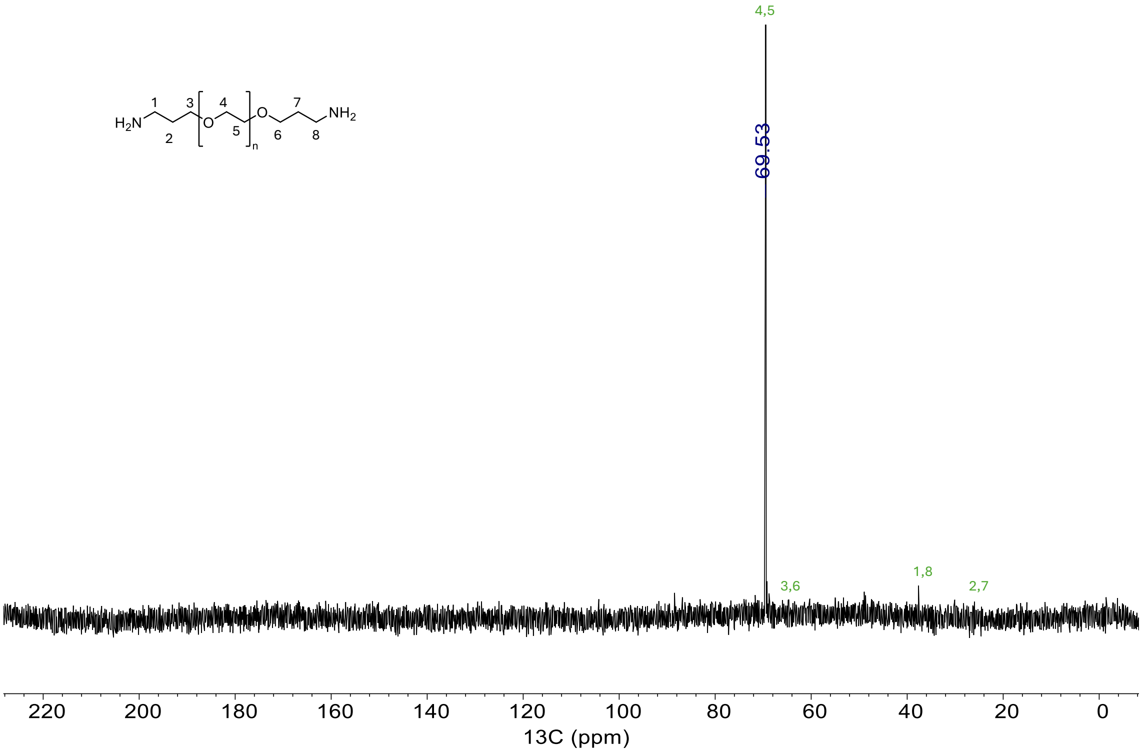

Figure S3. $^{13}C\{^{1}H\}$ NMR (126 MHz, D_2O) spectrum of the starting material PEG diamine, Mn=1500.

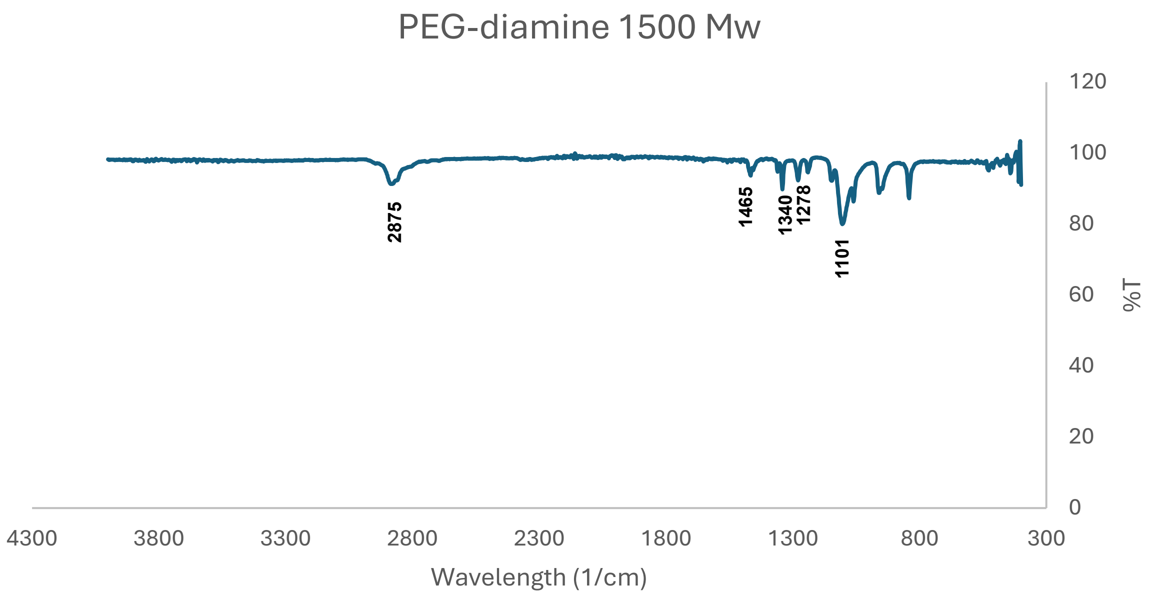

Figure S4. ATR-FTIR spectrum of the starting material PEG diamine, Mn=1500.

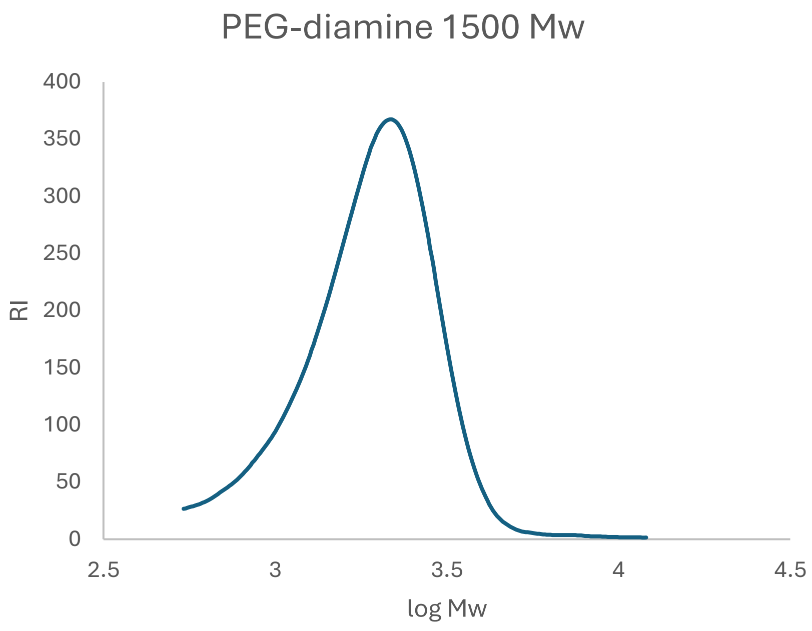

Figure S5. GPC data of the starting material PEG diamine, Mn=1500.

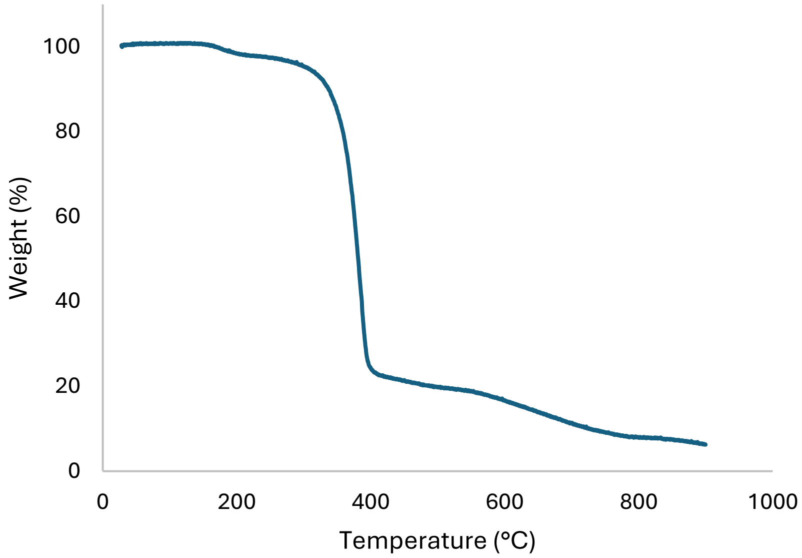

Figure S6. TGA Profile of the starting material PEG diamine, Mn=1500.

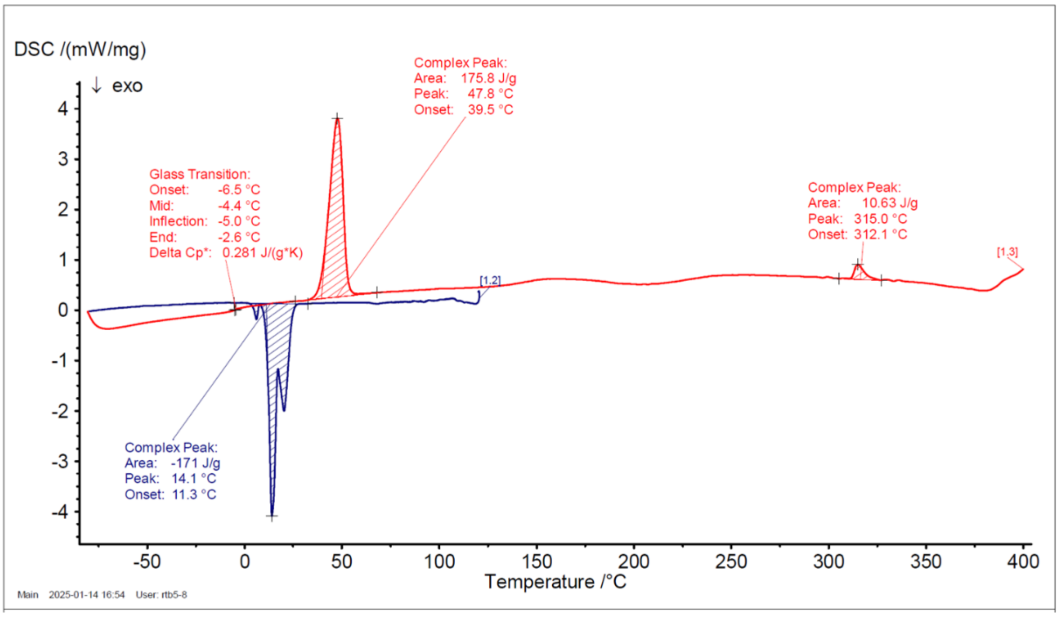

Figure S7. DSC trace of the starting material PEG diamine, Mn=1500.

5.2. Characterisation Details for the Isolated Polyurea PEU1

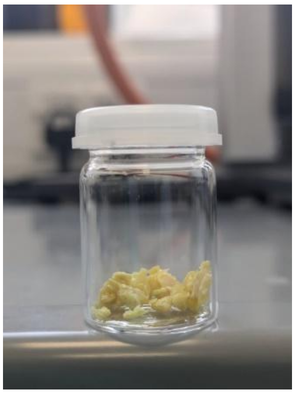

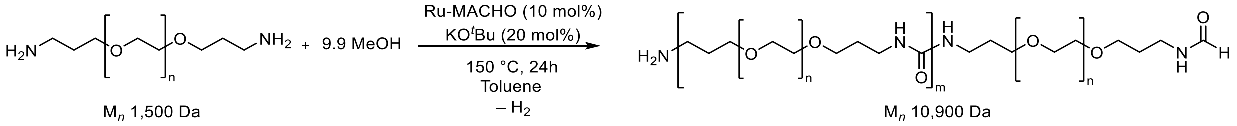

Yield= 61%

^{1}H NMR (500 MHz, DMSO-d_6): δH 1.24 (s, H-24), 1.56-1.68 (m, H-3,10,17), 2.17 (s, H-25), 3.11-3.18 (m, H-4,9,18,23), 3.38 (s, H-6,7,20,21), 3.52 (s, H-2,11,16), 5.82 (t, H-12, 14), 8.00 (s, H-26,28)

^{13}C{^{1}H} NMR (126 MHz, DMSO-d_6): δC 29.85 (C-3,10,17,24), 30.68 (C-2,11,16), 37.03 (C-25), 70.25 (C-4,6,7,9,18,20,21,23), 158.56 (C-13), 161.45 (C-13) 169.52 (C-27).

IR (ATR-FTIR, cm^{-1}): v 2885 (N-H, stretch), 1676 (C=O, stretch), 1558 (N-H, bend), 1350,1274 (C-N, stretch),1101(C-O, stretch) .

GPC: Mn =10900 Da (Đ:1.4)

TGA Profile: T_{donset}= 238°C, $T_{d,\ 5\%=}$ 330°C

DSC trace: = Tm=47.3 °C, Tc= 3.1 °C

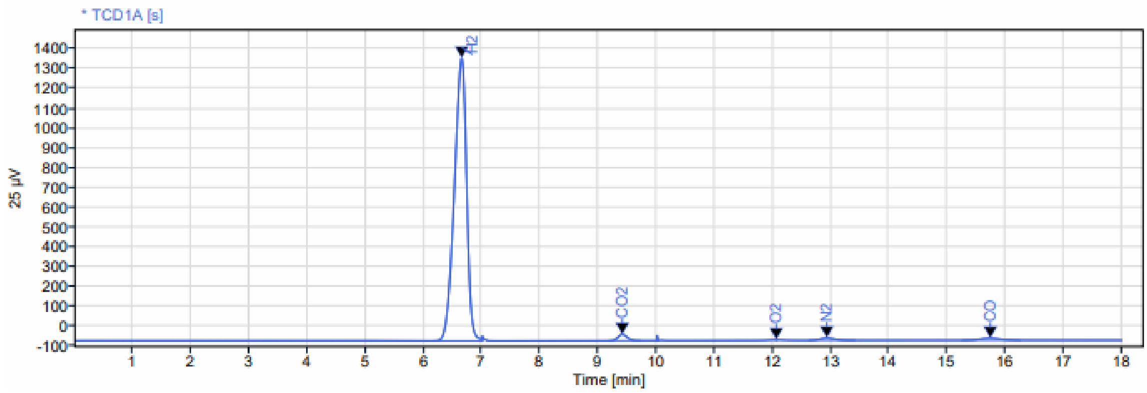

Signal:	* TCD1A [s]					
RT [min]	Type	Width [min]	Area	Height	Area%	Name
6.650	BV m	0.86	20952.79	1434.94	96.38	H2
9.411	VV	0.56	348.98	31.20	1.61	CO2
12.058	VV	0.04	5.07	2.41	0.02	O2
12.927	VV	0.81	194.28	11.19	0.89	N2
15.737	VV	1.01	239.26	9.91	1.10	CO
		Sum	21740.38			

Figure S8. GC-TCD analysis of the gas released from the reaction conducted as per the method described in PEU1.

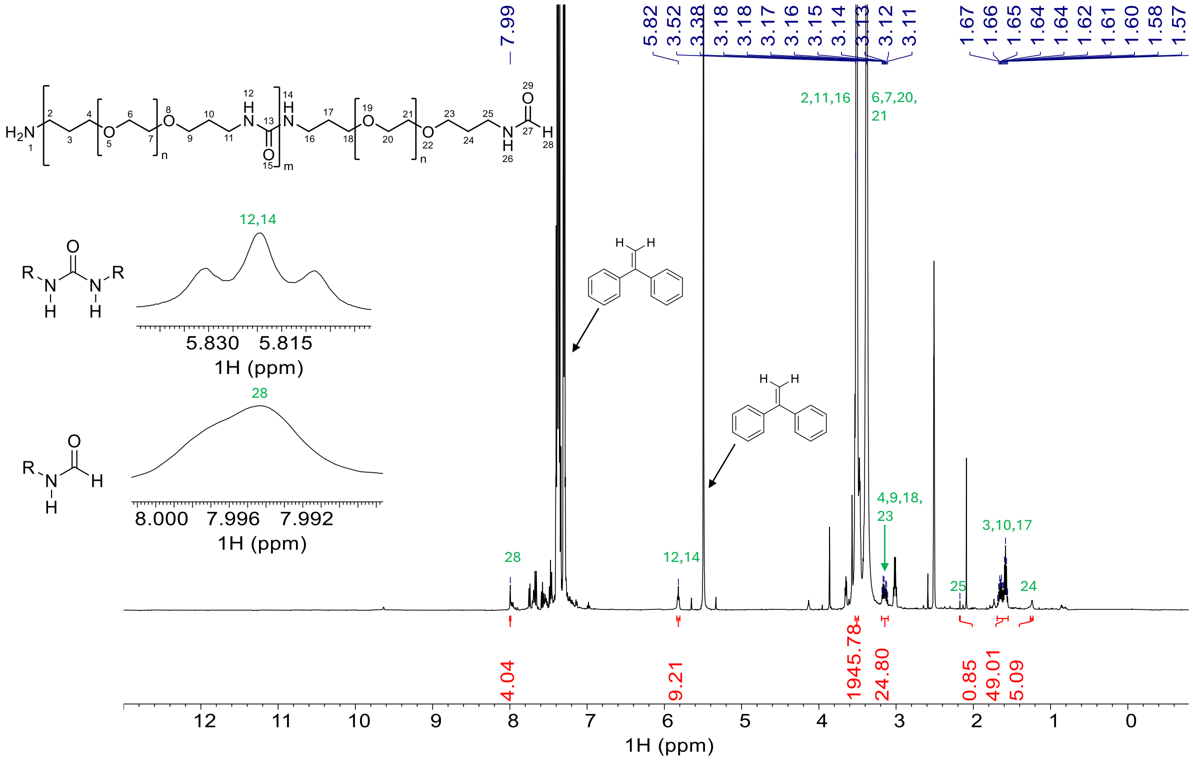

Figure S9. ^{1}H NMR (500 MHz, DMSO-d_6) spectrum of the isolated product from the polymerisation process conducted as per the method described in PEU1. 1,1-diphenylethylene is used as internal standard.

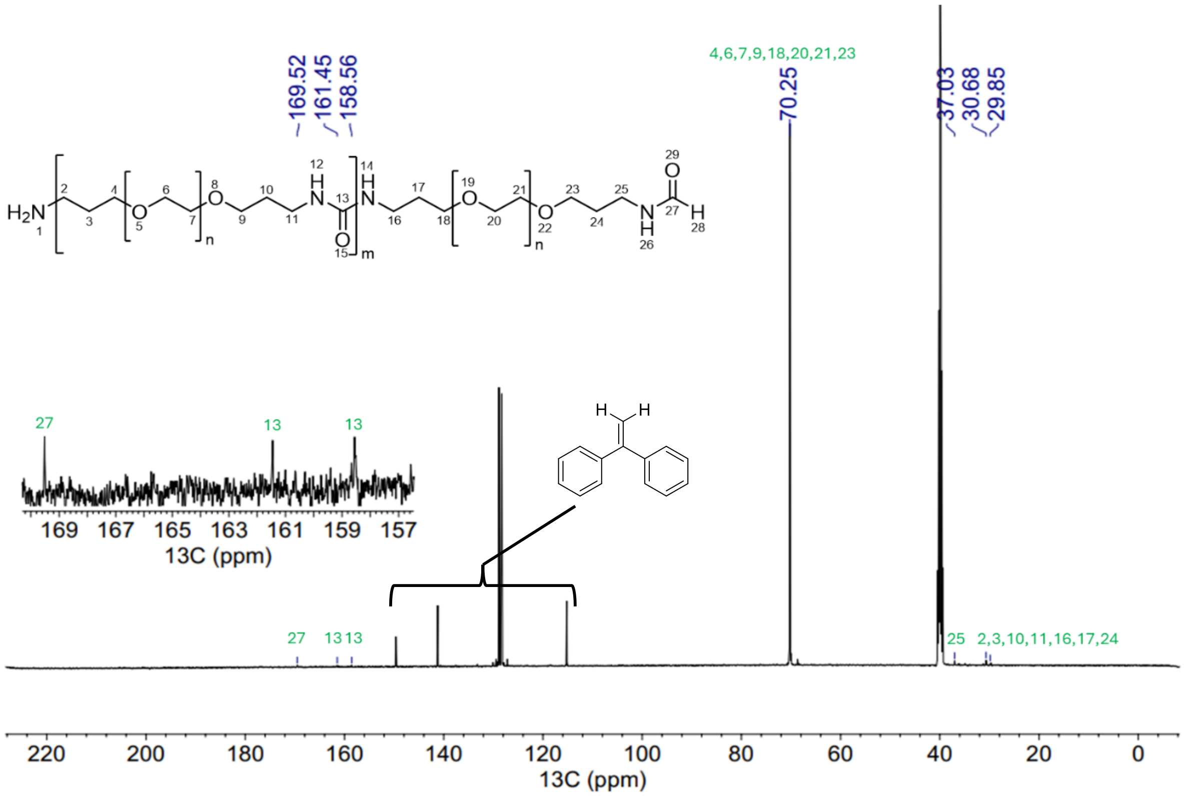

Figure S10. $^{13}C\{^{1}H\}$ NMR (126 MHz, DMSO-d_6) spectrum of the isolated product from the polymerisation process conducted as per the method described in PEU1. 1,1-diphenylethylene is used as internal standard.

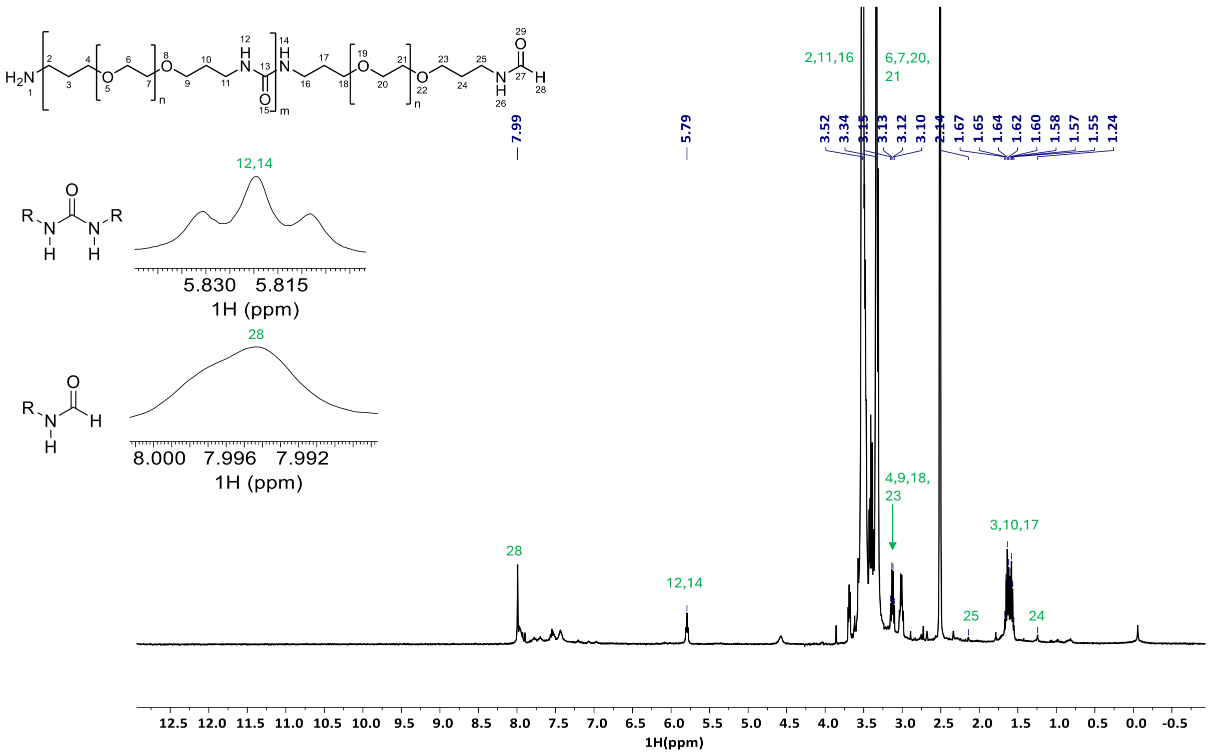

Figure S11. ^1H NMR (500 MHz, DMSO-d$_6$) spectrum of the isolated product from the polymerisation process conducted as per the method described in PEU1.

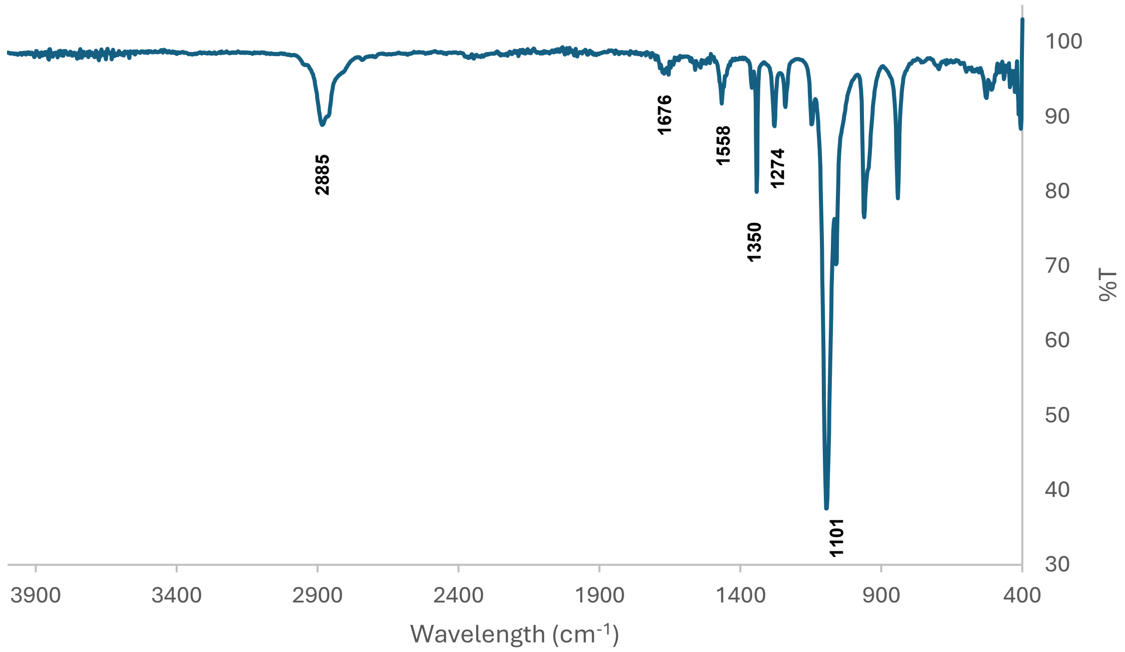

Figure S12. ATR-FTIR spectrum of the isolated product from the polymerisation process conducted as per the method described in PEU1.

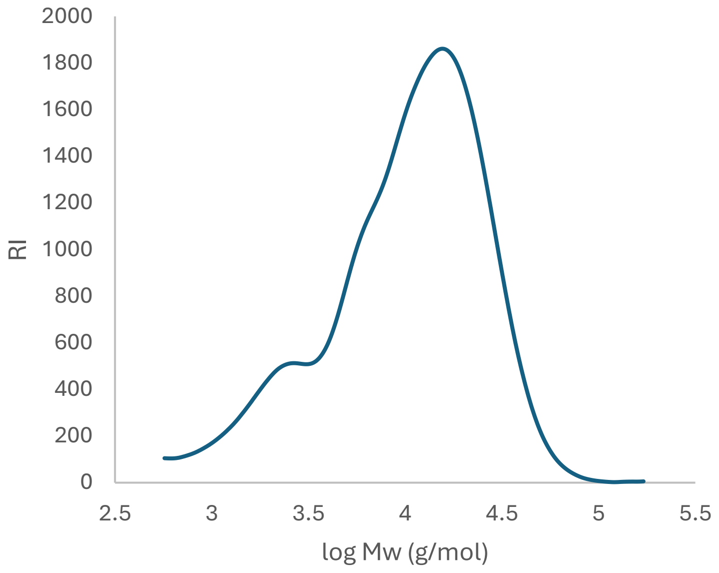

Figure S13. GPC data of the isolated product from the polymerisation process conducted as per the method described in PEU1.

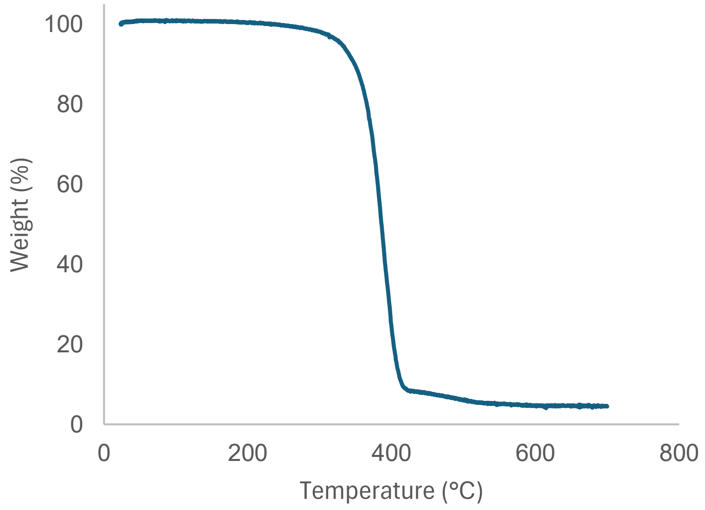

Figure S14. TGA Profile of the isolated product from the polymerisation process conducted as per the method described in PEU1.

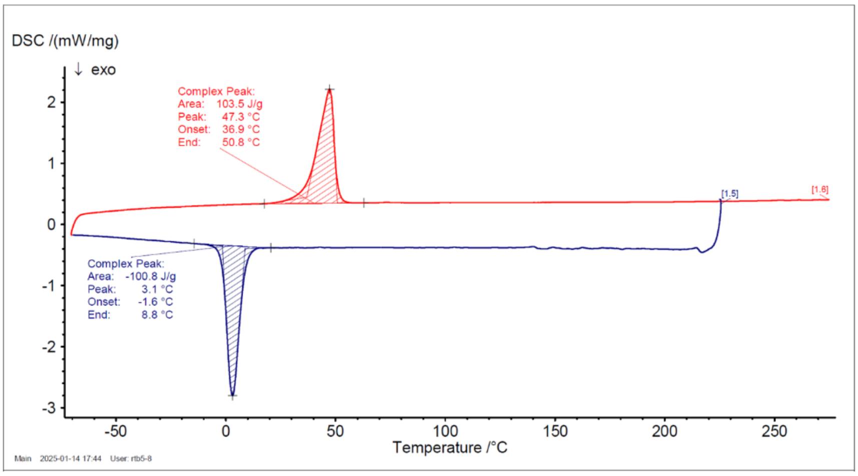

Figure S15. DSC trace of the isolated product from the polymerisation process conducted as per the method described in PEU1.

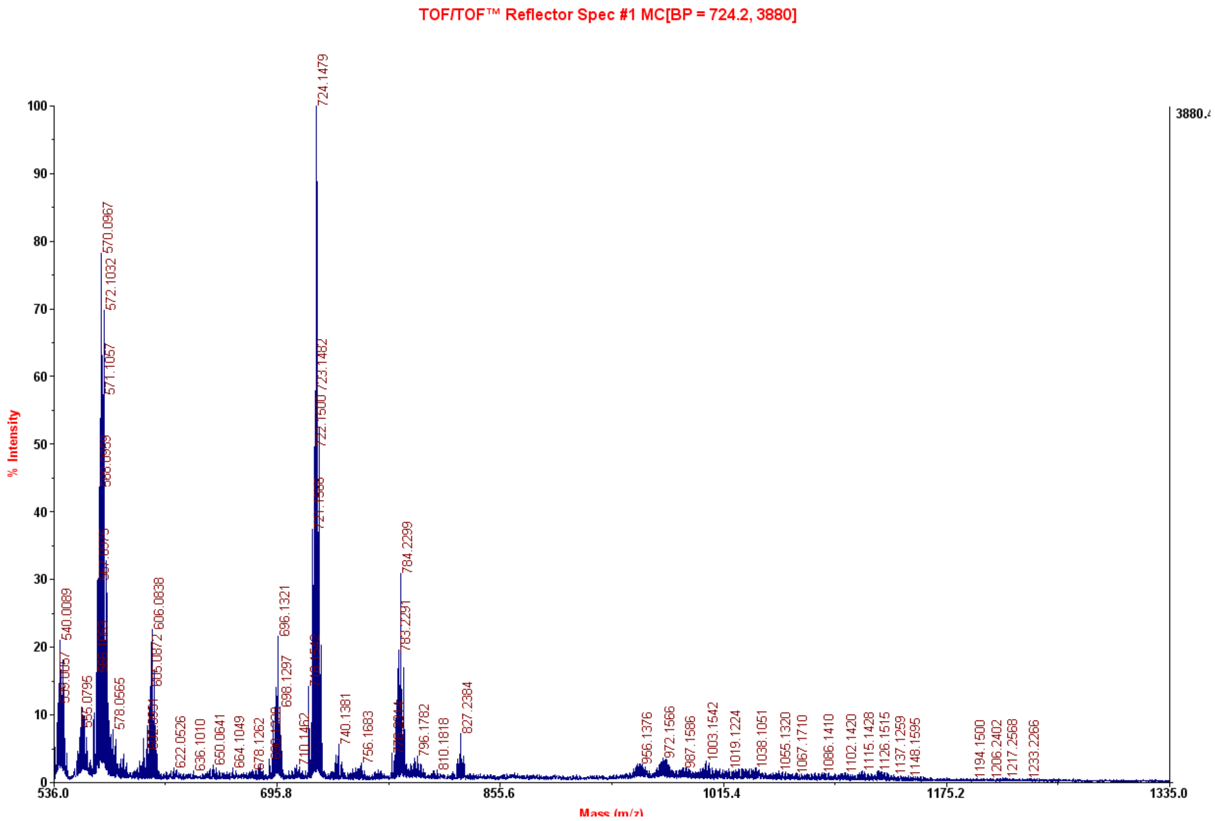

Figure S16. MALDI-TOF mass spectrometry of the isolated product from the polymerisation process conducted as per the method described in PEU1.

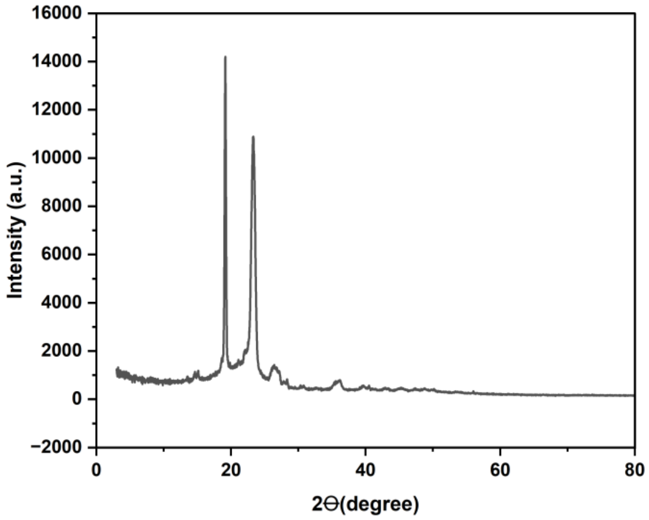

Powder XRD data of PEU1:

The polyetherurea (PEU1) formed was found to be crystalline in nature with the following characteristics:

Space group: P21 / a

Lattice parameters:

a = 8.0520 Å

b = 13.0627 Å

c = 19.6492 Å

β = 125.31 deg

V = 1686.533 Å^3

Crystal size = 70 nm

Density: 1.28 g/cm^3

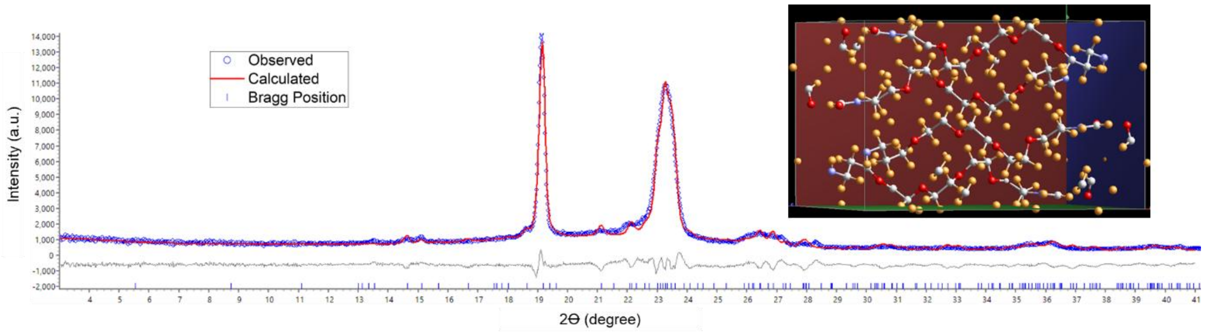

Figure S17. PXRD analysis of the isolated product from the polymerisation process conducted as per the method described in PEU1.

5.3. Characterisation Details for the Isolated Polyurea PEU2

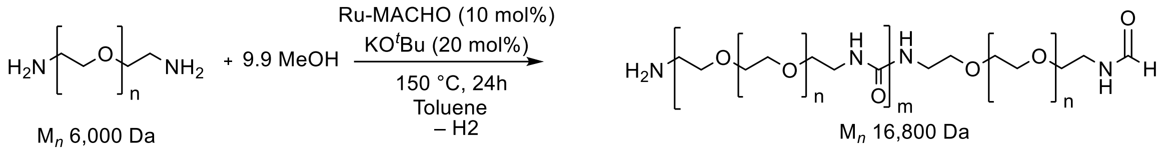

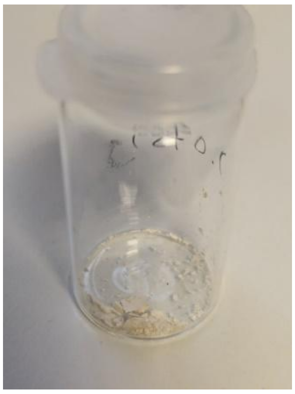

Yield= 66%

^{1}H NMR (500 MHz, DMSO-d$_6$): δH 1.23 (s, H-1), 2.51 (m, H-2,9,13,20), 3.47 (s, H-5,6,16,17), 3.50 (s, H-3,8,14,19), 5.99 (t, H-10,12), 7.97 (s, H-21, 23)

^{13}C{^{1}H} NMR (126 MHz, DMSO-d$_6$): δC 70.2 (C-3,5,6,8,14,16,17,19), 115.2, 127.2-130.1, 141.2, 149.6.

IR (ATR-FTIR, cm^{-1}): ν 2856 (N-H, stretch), 1624 (C=O, stretch), 1560 (N-H, bend), 1340,1278 (C-N, stretch),1095(C-O, stretch

GPC: Mn =16800 Da(Đ:2.0)

TGA Profile: T_{donset}= 329°C, $T_{d, 5\%=}$ 357°C

DSC trace: Tm=59 °C, Tc= 29.8 °C

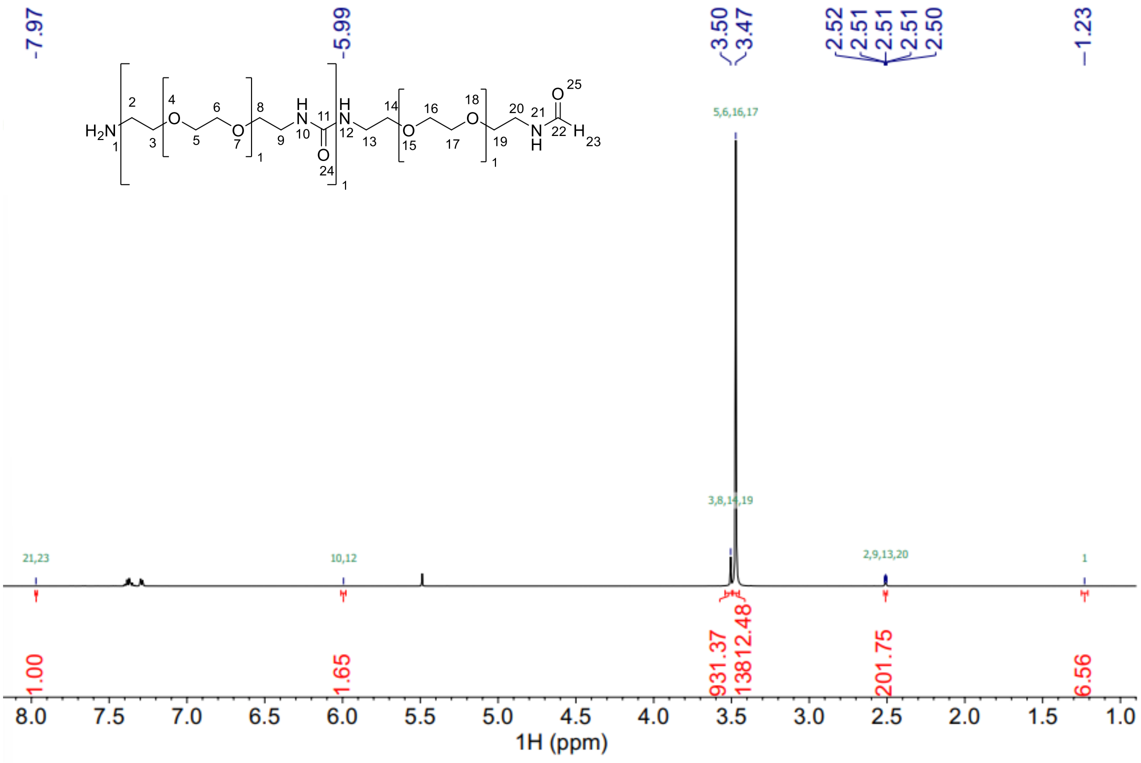

Figure S18. ^{1}H NMR (500 MHz, DMSO-d$_6$) spectrum of the isolated product from the polymerisation process conducted as per the method described in PEU2.

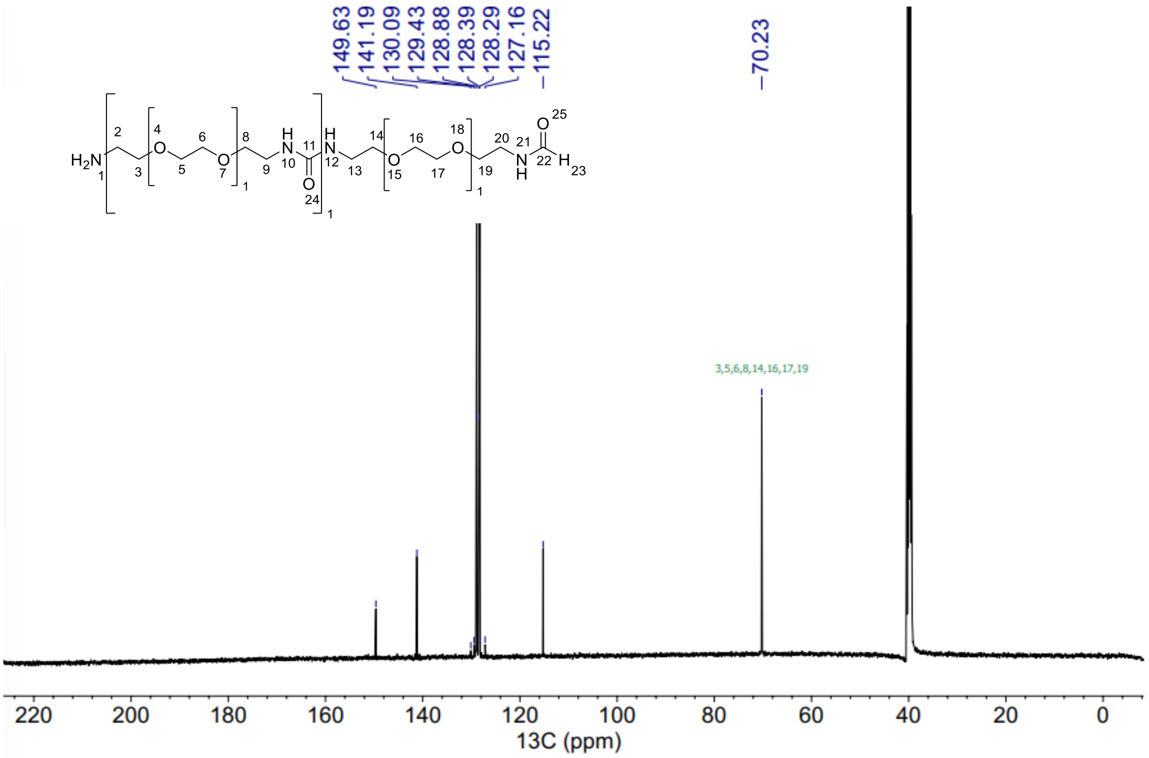

Figure S19. $^{13}C\{^{1}H\}$ NMR (126 MHz, DMSO-d_6) spectrum of the isolated product from the polymerisation process conducted as per the method described in PEU2.

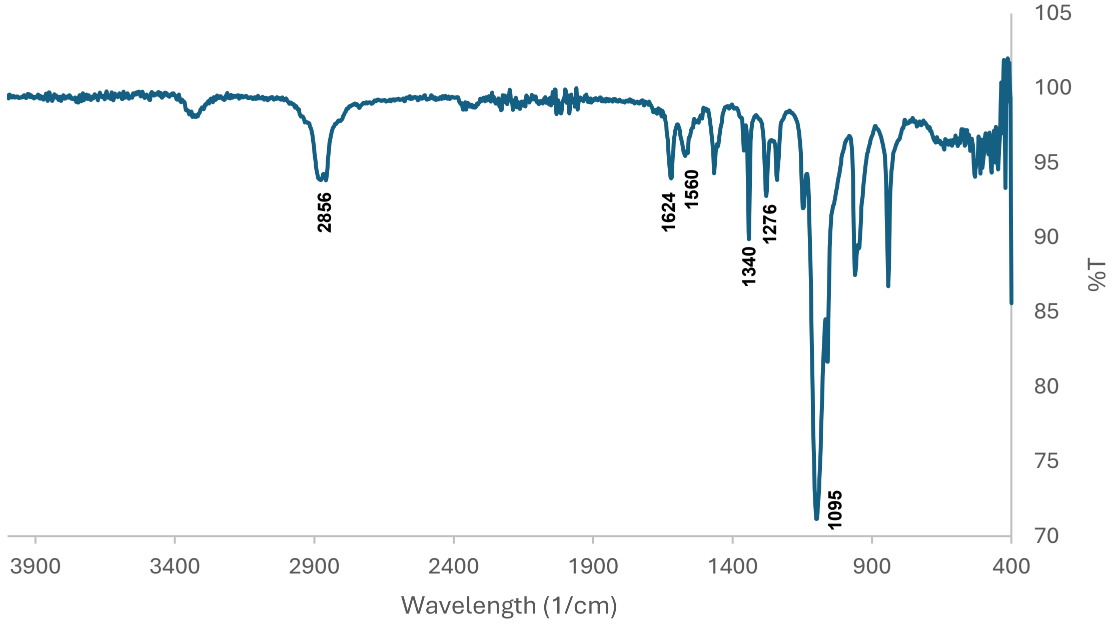

Figure S20. ATR-FTIR spectrum of the isolated product from the polymerisation process conducted as per the method described in PEU2.

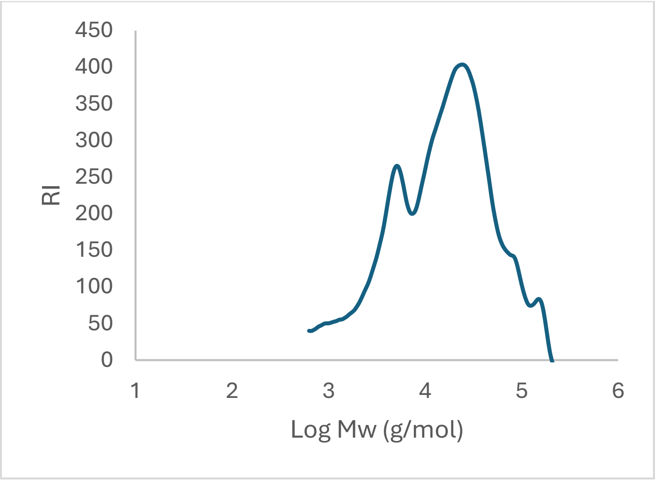

Figure S21. GPC data of the isolated product from the polymerisation process conducted as per the method described in PEU2.

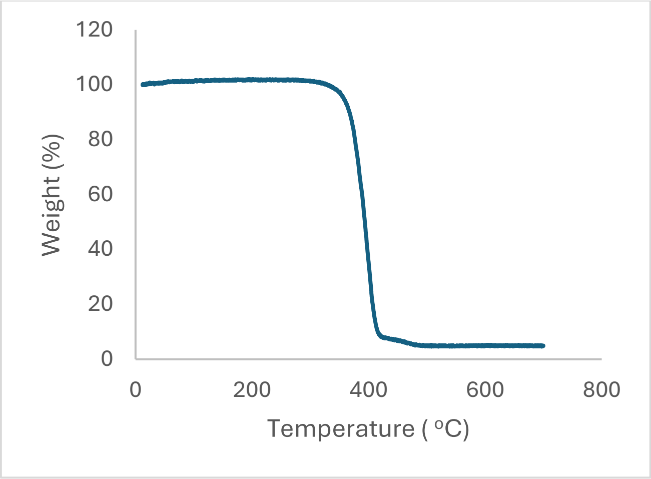

Figure S22. TGA Profile of the isolated product from the polymerisation process conducted as per the method described in PEU2.

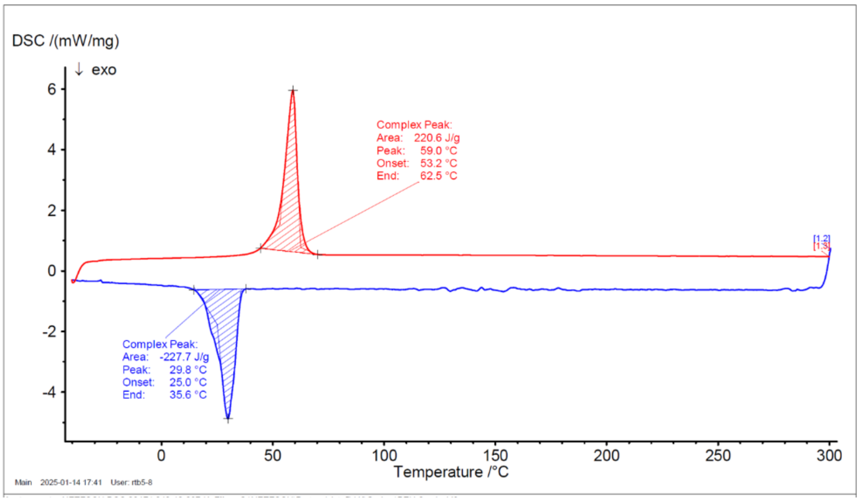

Figure S23. DSC trace of the isolated product from the polymerisation process conducted as per the method described in PEU2.

6. Procedure for making coin cells

The polyetherureas were mixed with deionised water to make a solution in a 1:45 ratio. This solution was then utilised in making slurries for binders where the ratio for the active material: binders: conductive carbon was in an 80:10:10 weight ratio. Some of the polyureas were mixed with SBR to make a composite so the binder was in a 1:1 ratio of polyurea:SBR emulating the industrially used CMC/SBR composites. Before this, the conductive carbon and active material were ground for 15 minutes using a pestle and mortar and then added to the different binder solutions. The slurry was stirred overnight to ensure good homogeneity and cast onto Aluminium foil (Advent Ltd.) using a doctor blade at 200 μm. This cast was allowed to air dry for 30 minutes and the electrodes were punched out in 13 mm diameter circles. The electrodes were dried in a glovebox antechamber at 80°C

overnight in dynamic vacuum. The coin cells were assembled in an M-Braun glovebox under an argon atmosphere (H_2O<0.1 ppm, O_2<0.1 ppm). The electrolyte (80-100 μL) used was 1M $LiPF_6$ in ethylene carbonate/dimethyl carbonate (1:1) (BASF) with glass fibre separator (Whatman, GF/F) and lithium metal as the counter electrode. The approximate active mass loading for CMC, PEU1 and PEU1+SBR are 2.71, 2.73, and 2.25 mg cm^{-2}, respectively.

7. Galvanostatic Data

*Every binder has been tested 2 times to study reproducibility with their data as described below.

7.1. CMC

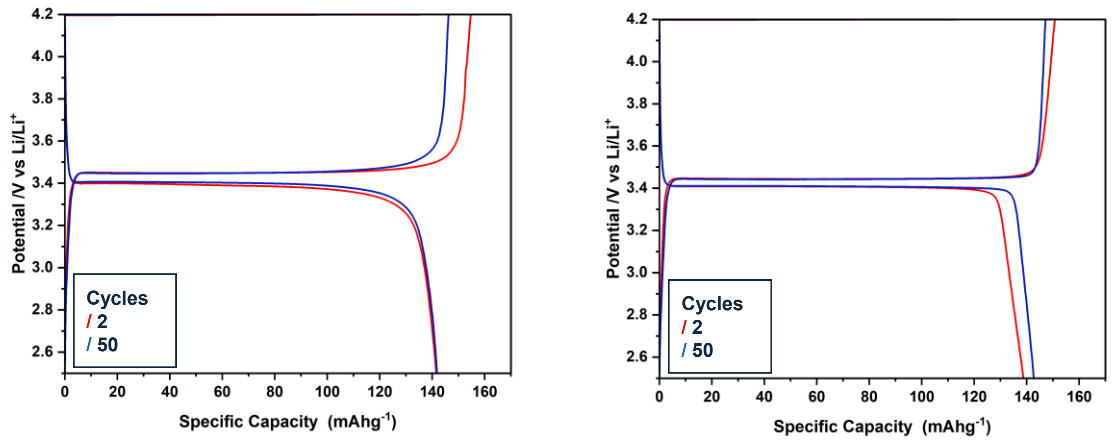

Figure S24. Specific capacity against potential graph of LFP in a Half cell with CMC as the binder with a 10 mAg^{-1} current density (0.06C current rate), cycled 50 times between a voltage window of 2.5V to 4.2V

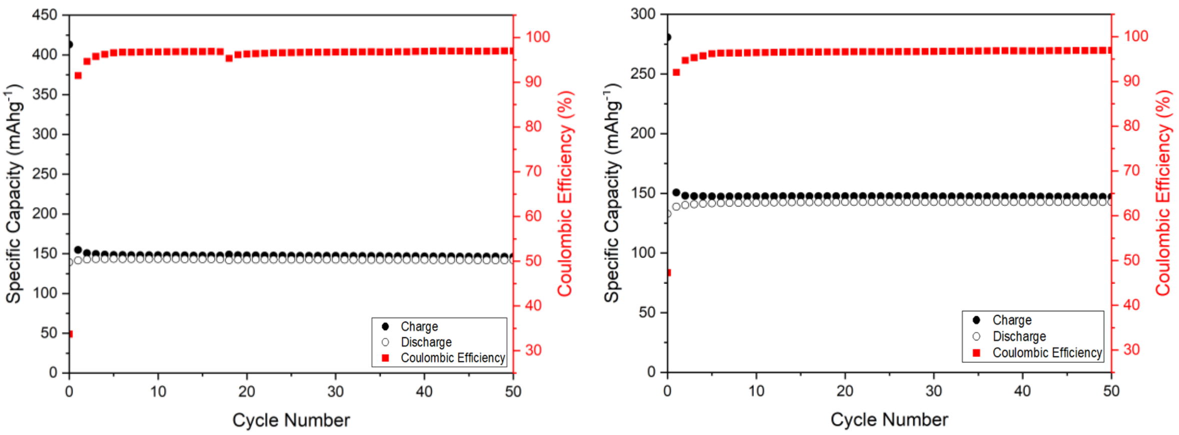

Figure S25. Specific capacity and coulombic efficiency of LFP in a Half Cell with CMC as the binder with a 10 mAg^{-1} current density (0.06C current rate), cycled 50 times between a voltage window 2.5V to 4.2V.

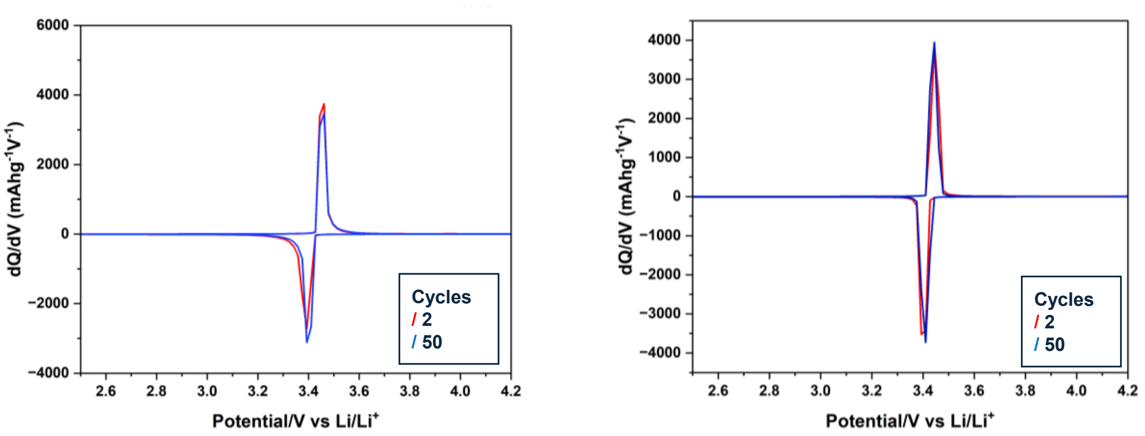

Figure S26. dQ/dV plot of LFP in a Half cell CMC as the binder at 10 mAg^{-1} current density (0.06C current rate), cycled 50 times between a voltage window 2.5V to 4.2V.

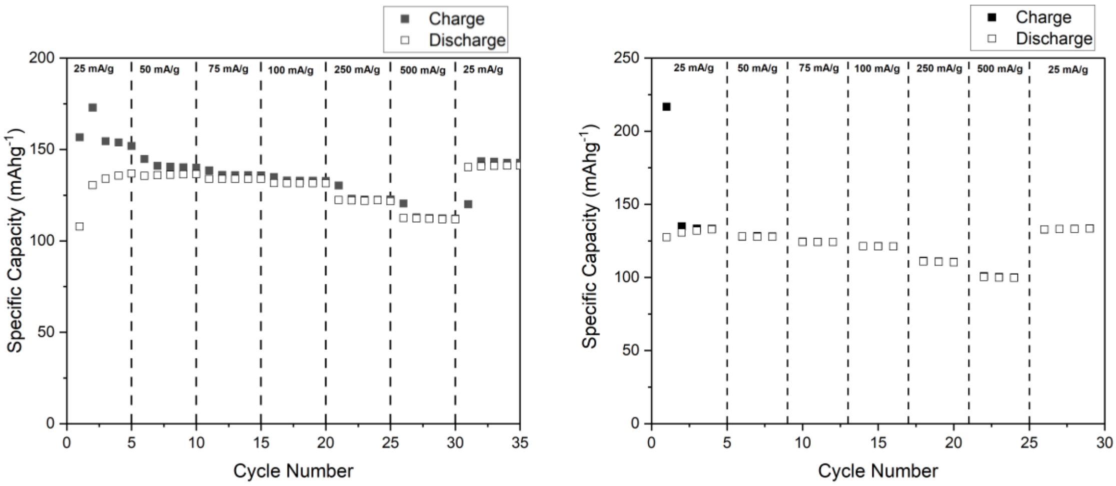

Figure S27. Rate performance of LFP in a Half cell with CMC as a binder at current rate ranging from 25 mAg^{-1} to 500 mAg^{-1}, cycled 5 times at each current density, between a voltage window 2.5V to 4.2V.

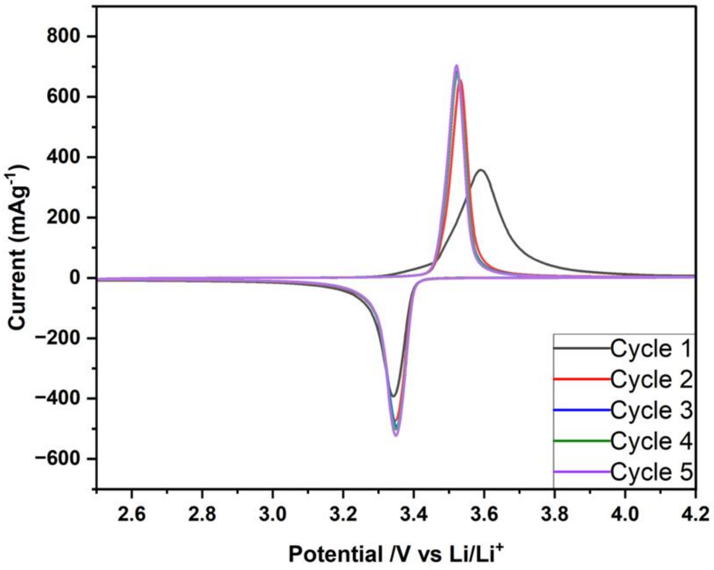

Figure S28. Cyclic voltammogram curves of Half-cell with CMC, cycled at 0.1 mVs^{-1} for 5 times between a voltage window 2.5V to 4.2V.

7.2. CMC with SBR

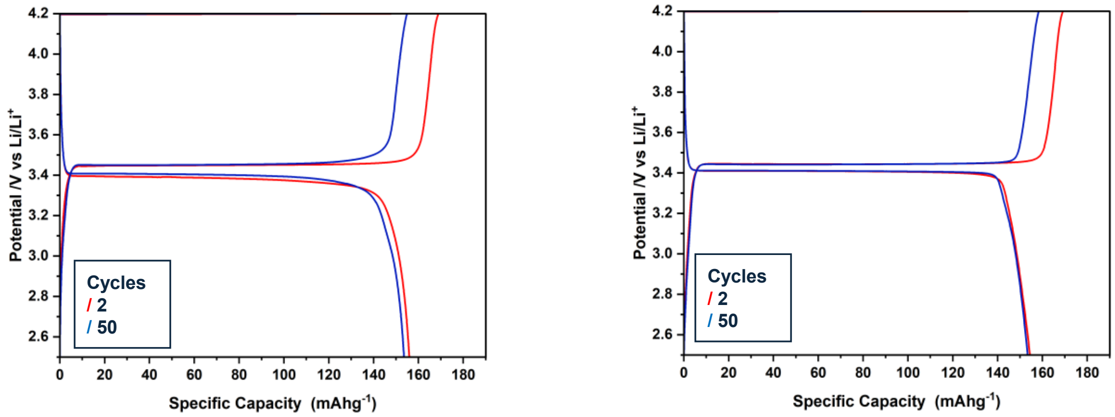

Figure S29. Specific capacity against potential graph of LFP in a Half cell with CMC with SBR in ratio 50:50 as the binder at 10 mAg^{-1} current density (0.06C current rate), cycled 50 times between a voltage window 2.5V to 4.2V.

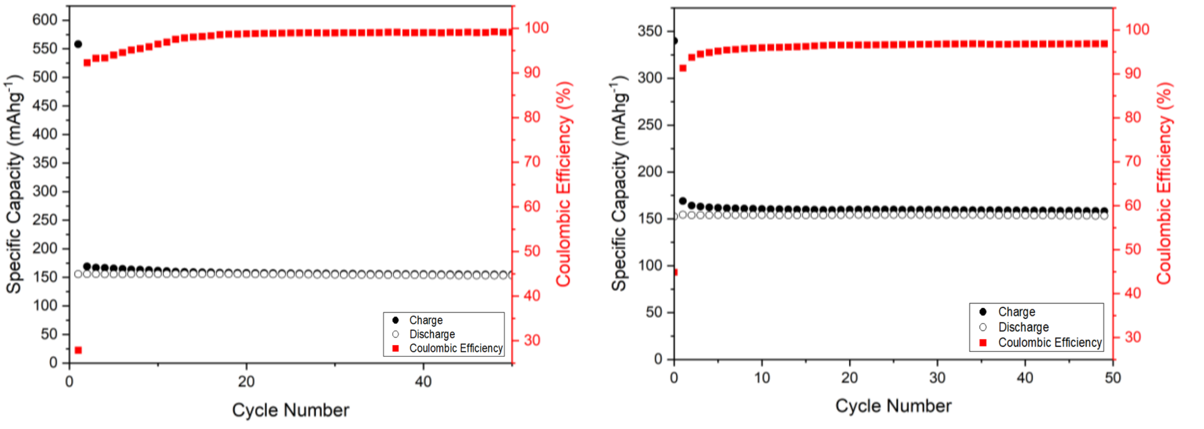

Figure S30. Specific capacity and coulombic efficiency of LFP in a Half cell with CMC with SBR as the binder at 10 mAg^{-1} current density (0.06C current rate), cycled 50 times between a voltage window 2.5V to 4.2V.

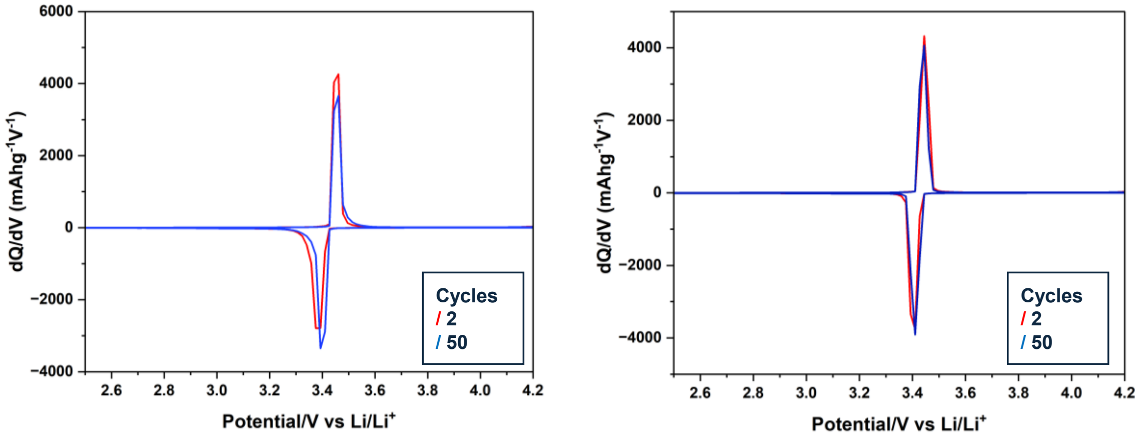

Figure S31. dQ/dV plot of LFP in a Half cell with CMC with SBR as the binder at 10 mAg^{-1} current density (0.06C current rate), cycled 50 times between a voltage window 2.5V to 4.2V.

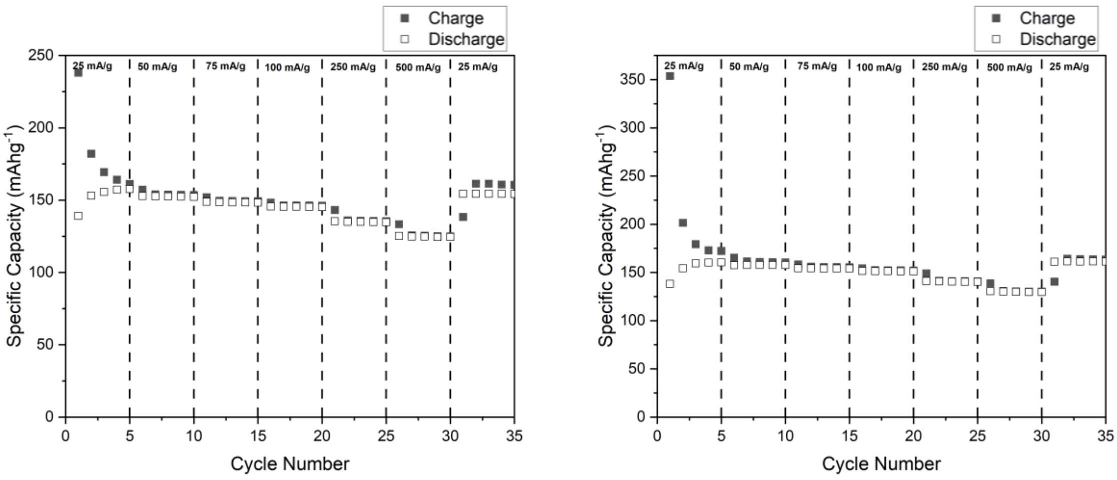

Figure S32. Rate performance of LFP in a Half cell with CMC with SBR as the binder at current rate ranging from 25 mAg^{-1} to 500 mAg^{-1}, cycled 5 times at each current density, between a voltage window 2.5V to 4.2V.

7.3. PEG diamine, Mn=1500

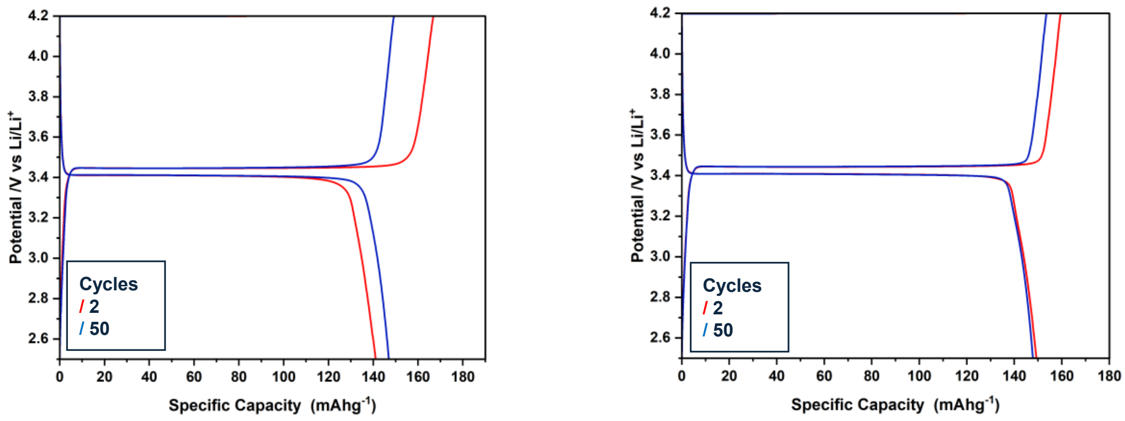

Figure S33. Specific capacity against potential graph of LFP in a Half cell with PEG Diamine as the binder, Mn=1500 with a 10 mAg^{-1} current density (0.06C current rate), cycled 50 times between a voltage window 2.5V to 4.2V.

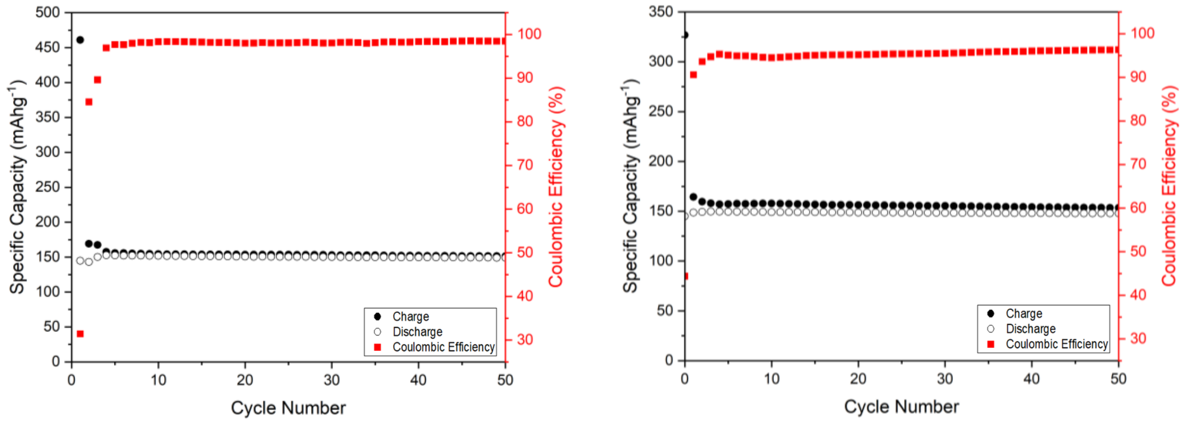

Figure S34. Specific capacity and coulombic efficiency of LFP in a Half cell with PEG Diamine as the binder, Mn=1500 with a 10 mAg^{-1} current density (0.06C current rate), cycled 50 times between a voltage window 2.5V to 4.2V.

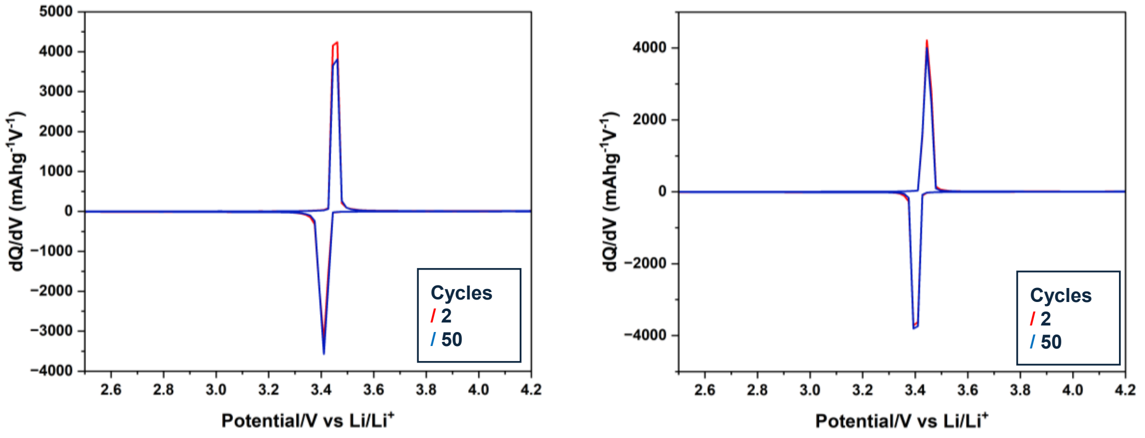

Figure S35. dQ/dV plot of LFP in a Half cell with PEG Diamine as the binder, Mn=1500 with a 10 mAg^{-1} current density (0.06C current rate), cycled 50 times between a voltage window 2.5V to 4.2V.

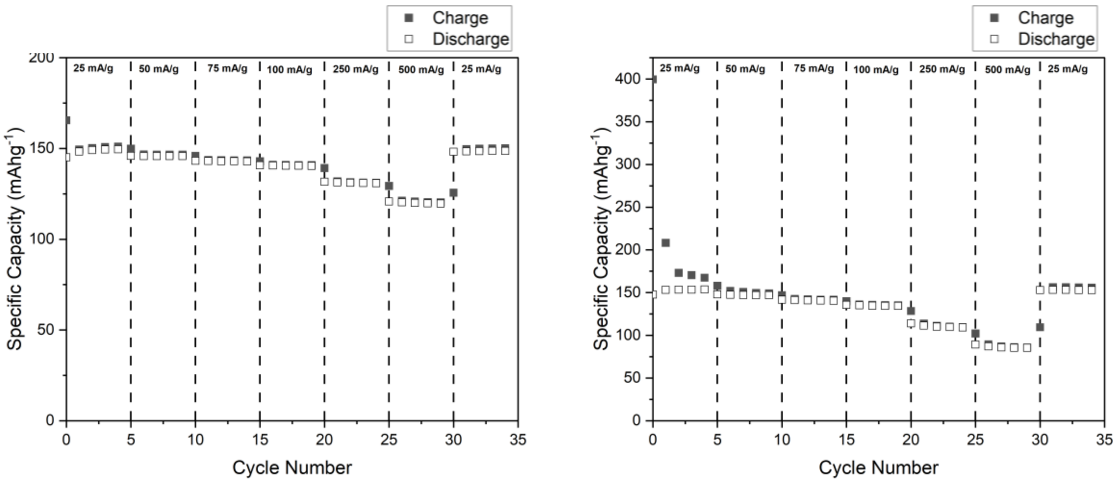

Figure S36. Rate performance of LFP in a Half cell with PEG Diamine as the binder, Mn=1500 from a current rate of 25 mAg^{-1} to 500 mAg^{-1}, cycled 5 times at each current density, between a voltage window 2.5V to 4.2V.

7.4. PEG Diamine, Mn=1500 with SBR

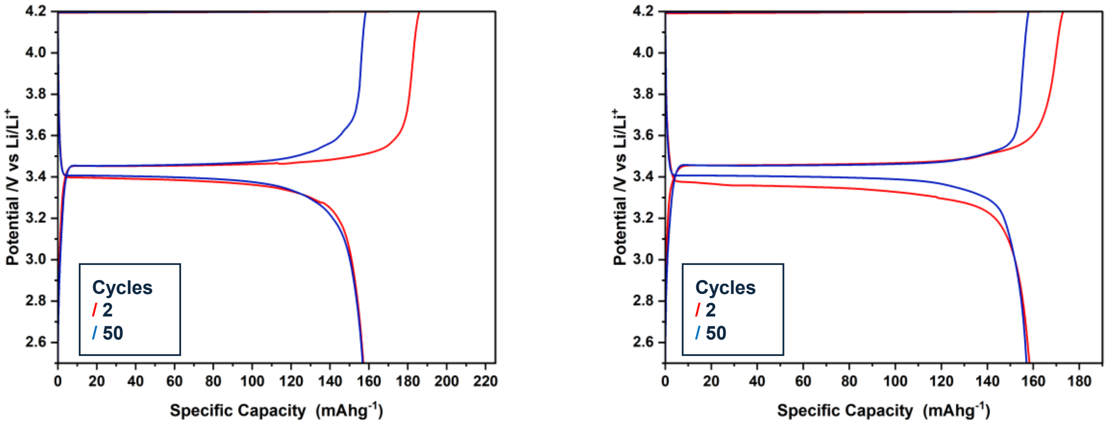

Figure S37. Specific capacity against potential graph of LFP in a Half cell with PEG diamine with SBR in ratio 50:50 as the binder with a 10 mAg^{-1} current density (0.06C current rate), cycled 50 times between a voltage window 2.5V to 4.2V.

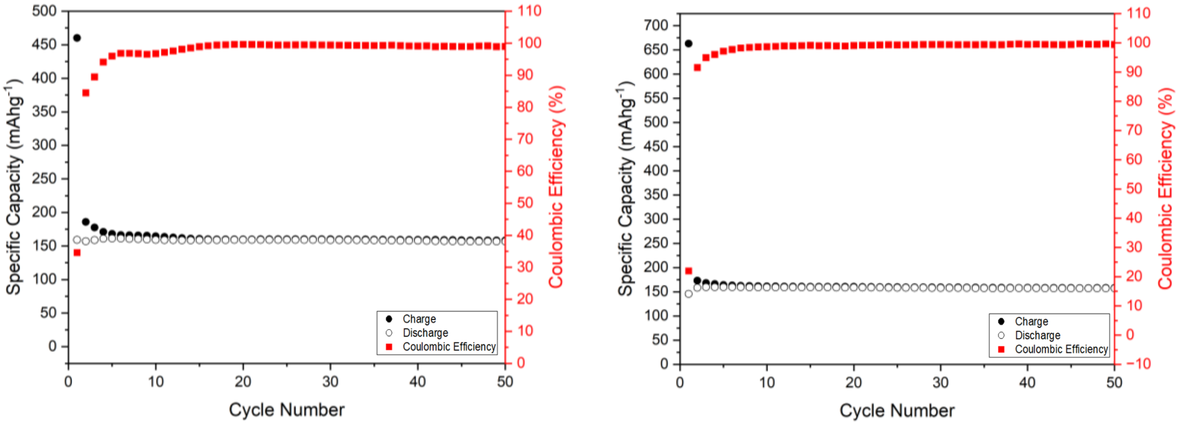

Figure S38. Specific capacity and coulombic efficiency of LFP in a Half Cell with PEG diamine with SBR as the binder with a 10 mAg^{-1} current density (0.06C current rate), cycled 50 times between a voltage window 2.5V to 4.2V.

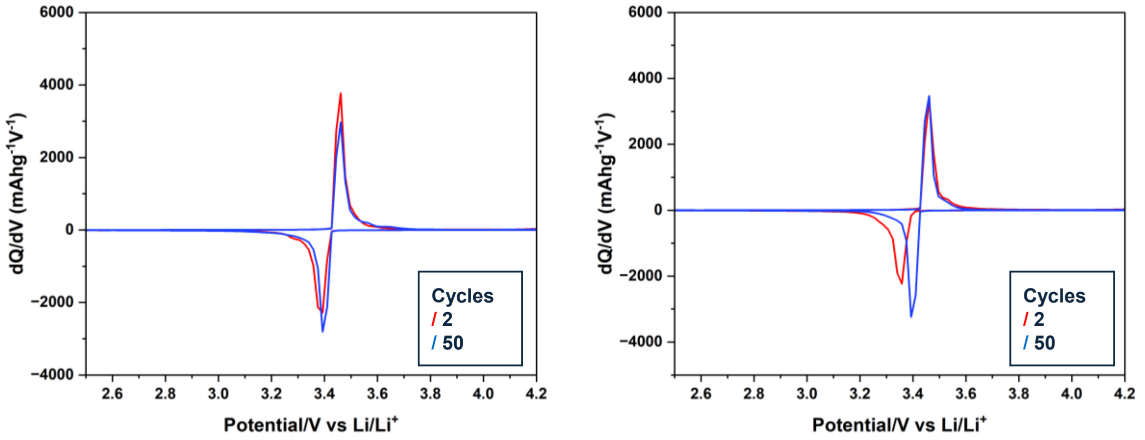

Figure S39. dQ/dV plot of LFP in a Half cell with PEG diamine with SBR as the binder at 10 mAg^{-1} current density (0.06C current rate), cycled 50 times between a voltage window 2.5V to 4.2V.

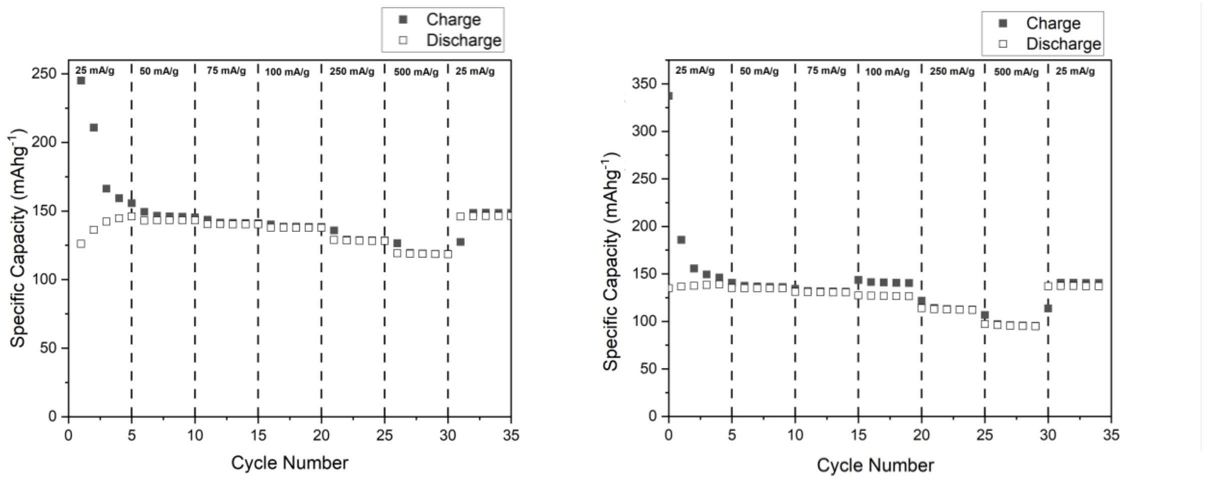

Figure S40. Rate performance of LFP in a Half cell with PEG Diamine with SBR as the binder at current rate ranging from 25 mAg^{-1} to 500 mAg^{-1}, cycled 5 times at each current density, between a voltage window 2.5V to 4.2V.

7.5. PEU1

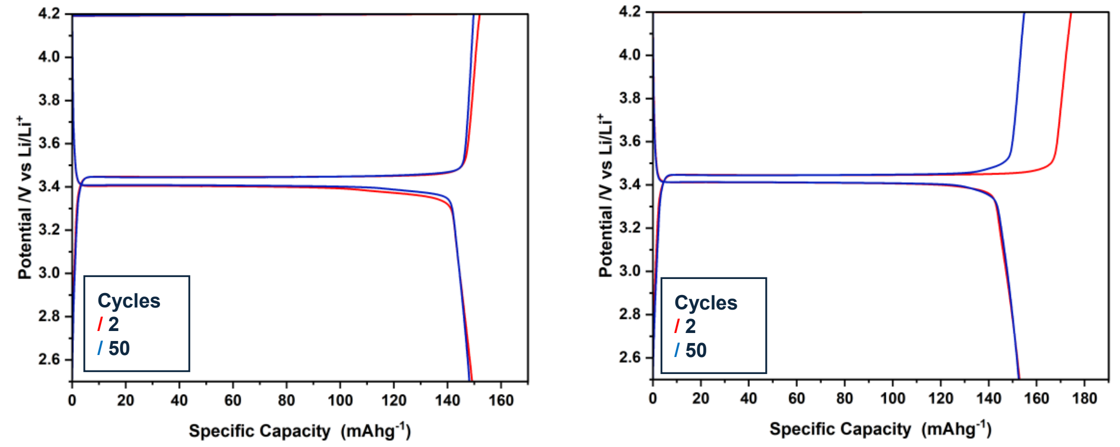

Figure S41. Specific capacity against potential graph of LFP in a Half cell with PEU1 as the binder at 10 mAg $^{-1}$current density (0.06C current rate), cycled 50 times between a voltage window 2.5V to 4.2V.

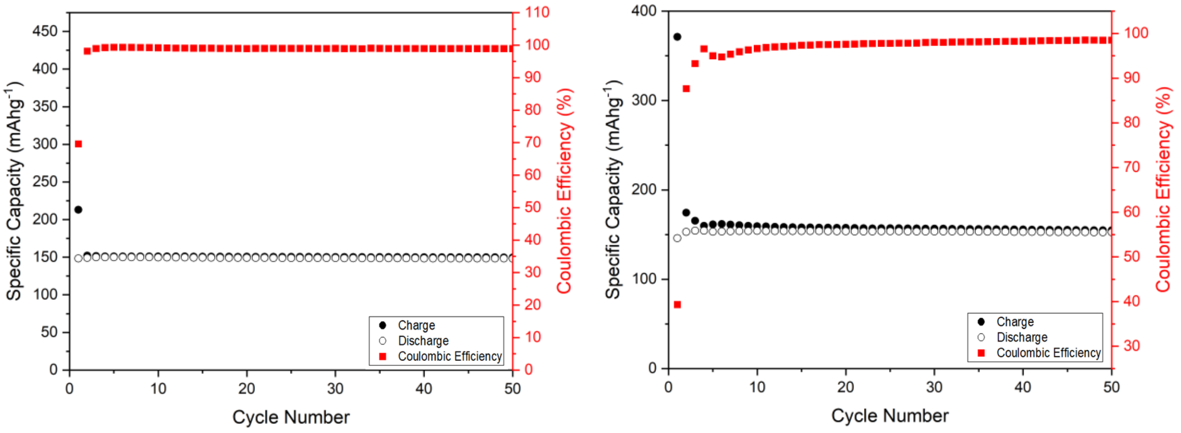

Figure S42. Specific capacity and coulombic efficiency of LFP in a Half cell with PEU1 as the binder at 10 mAg^{-1} current density (0.06C current rate), cycled 50 times between a voltage window 2.5V to 4.2V.

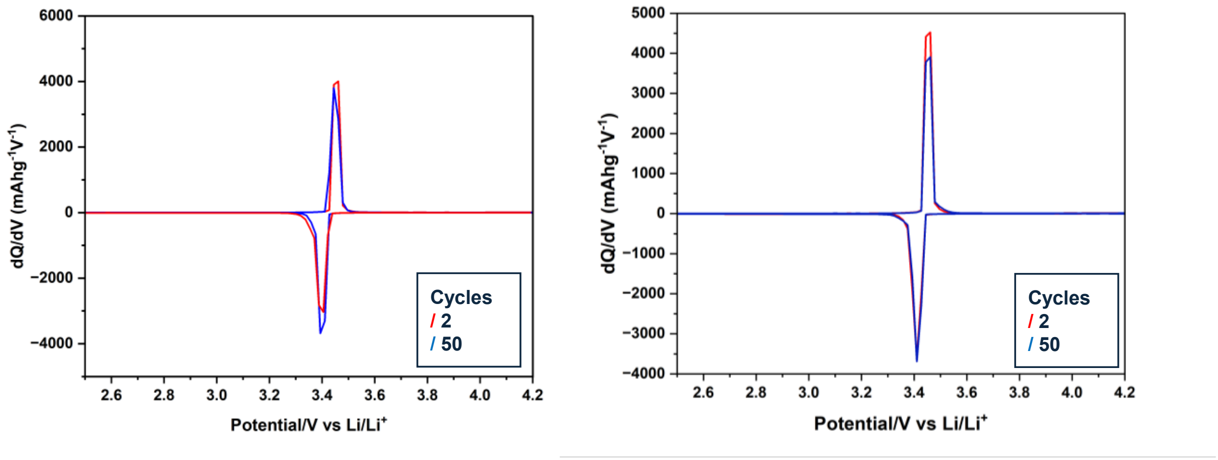

Figure S43. dQ/dV of LFP in a Half cell with PEU1 as the binder at 10 mAg^{-1} current density (0.06C current rate), cycled 50 times between a voltage window 2.5V to 4.2V.

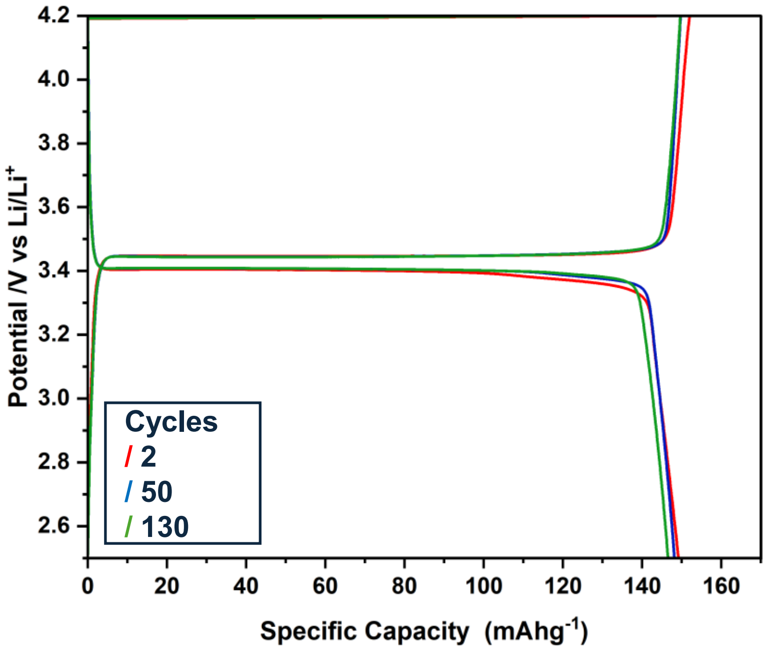

Figure S44. Specific capacity against potential graph of LFP in a Half cell with PEU1 as the binder at 10 mAg^{-1} current density (0.06C current rate), cycled 130 times between a voltage window 2.5V to 4.2V.

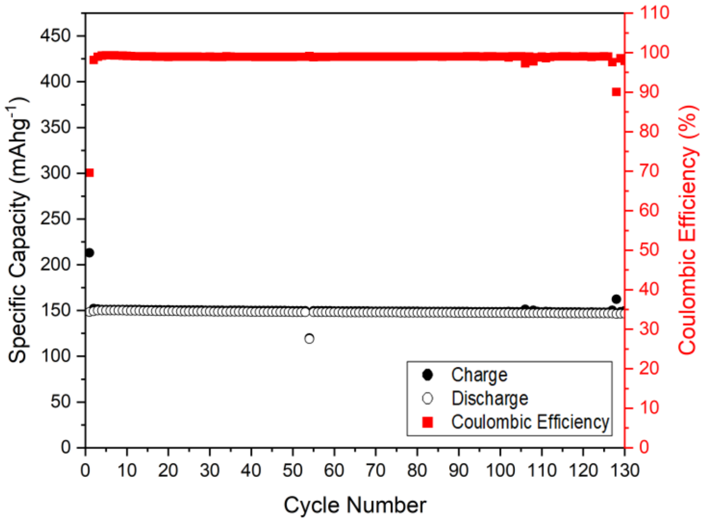

Figure S45. Specific capacity and coulombic efficiency of LFP in a Half cell with PEU1 as the binder at 10 mAg^{-1} current density (0.06C current rate), cycled 130 times between a voltage window 2.5V to 4.2V.

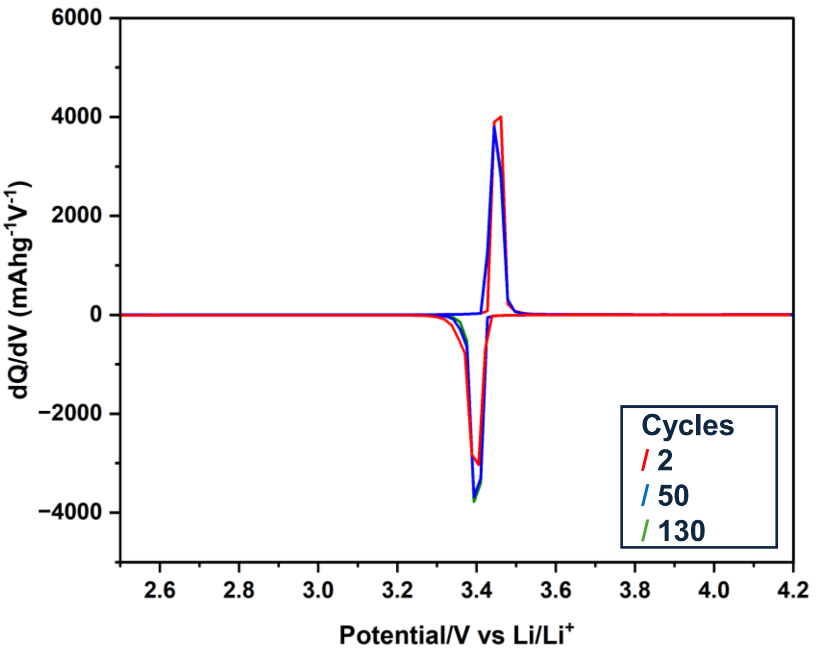

Figure S46. dQ/dV of LFP in a Half cell with PEU1 as the binder at 10 mAg^{-1} current density (0.06C current rate), cycled 130 times between a voltage window 2.5V to 4.2V.

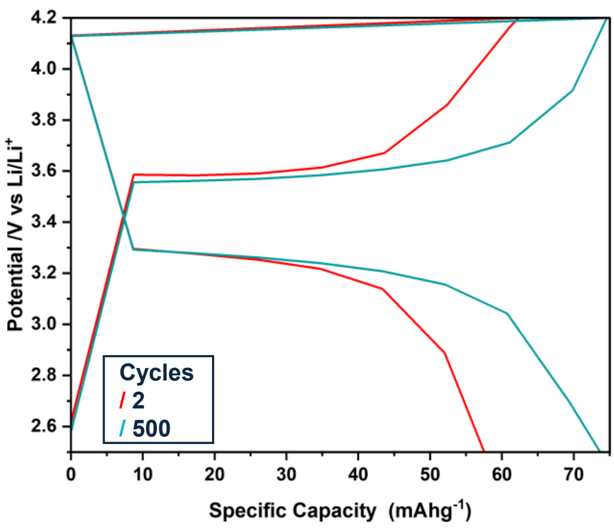

Figure S47. Specific capacity against potential graph of LFP in a Half cell with PEU1 as the binder at 3C current rate, cycled 500 times between a voltage window 2.5V to 4.2V.

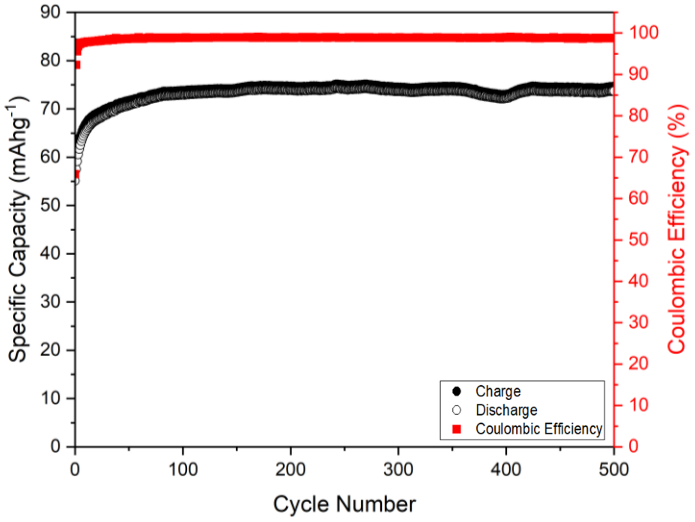

Figure S48. Specific capacity and coulombic efficiency of LFP in a Half cell with PEU1 as the binder at 3C current rate, cycled 500 times between a voltage window 2.5V to 4.2V.

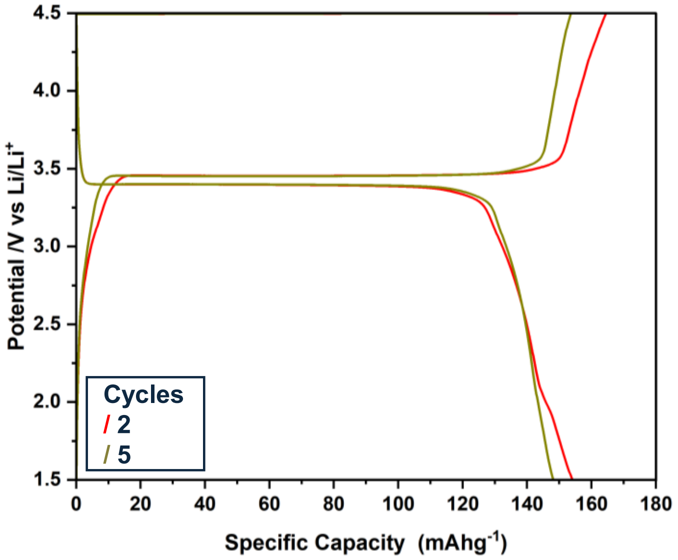

Figure S49. Specific capacity against potential graph of LFP in a Half cell with PEU1 as the binder at 10 mAg^{-1} current density (0.06C current rate), cycled 5 times between a voltage window 1.5V to 4.5V.

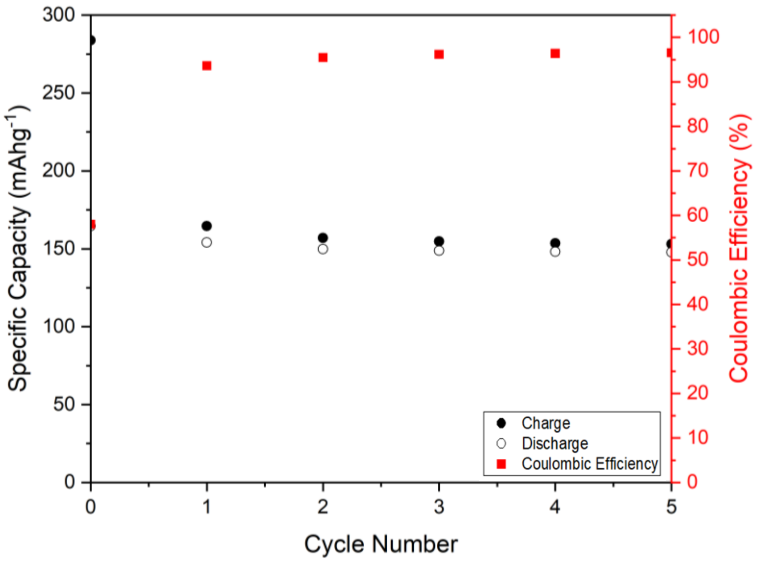

Figure S50. Specific capacity and coulombic efficiency of LFP in a Half cell with PEU1 as the binder at 10 mAg^{-1} current density (0.06C current rate), cycled 5 times between a voltage window 1.5V to 4.5V.

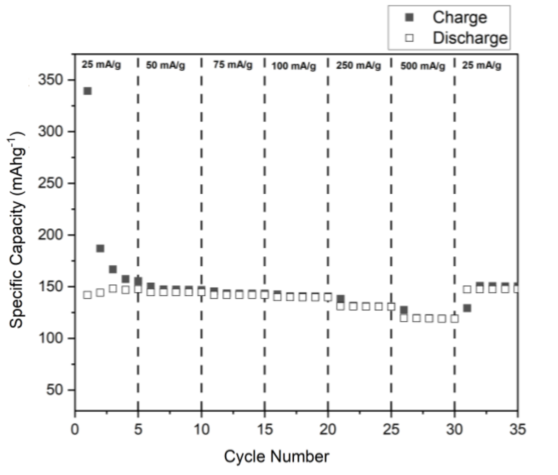

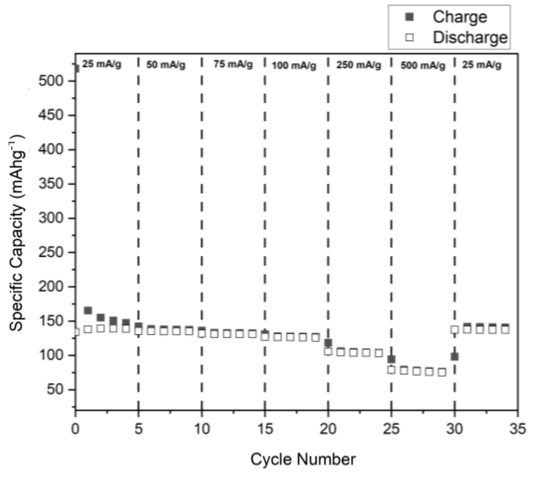

Figure S51. Rate performance of LFP in a Half cell with PEU1 as the binder at current rate ranging from 25 mAg^{-1} to 500 mAg^{-1}, cycled 5 times at each current density, between a voltage window 2.5V to 4.2V.

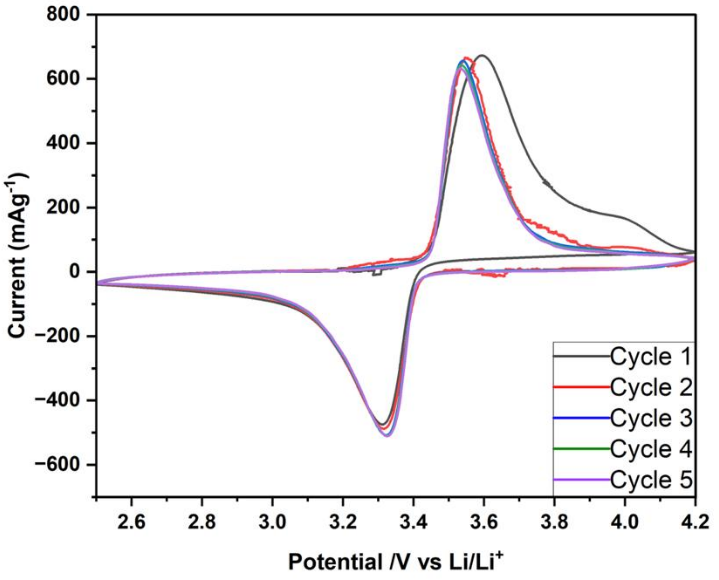

Figure S52. Cyclic voltammogram curves of Half cell with PEU1, cycled at 0.1 mVs^{-1} for 5 times between a voltage window 2.5V to 4.2V.

7.6. PEU1 with SBR

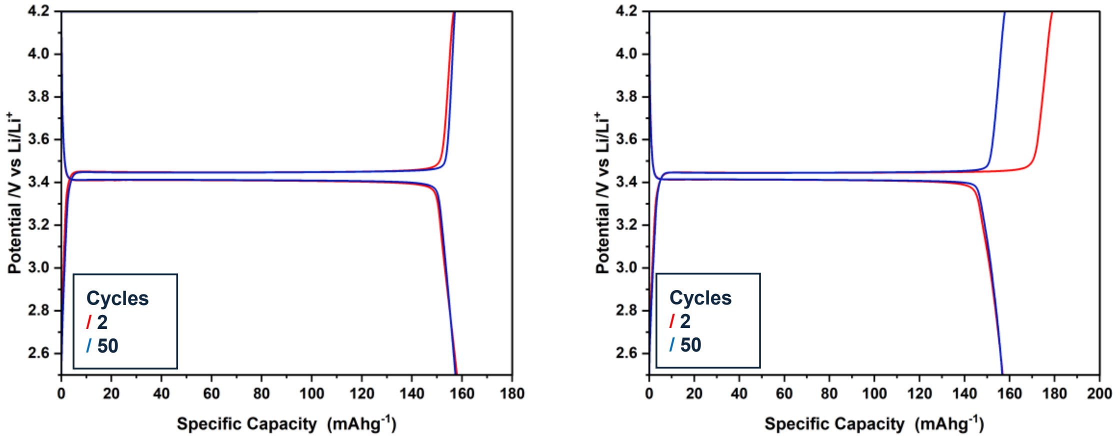

Figure S53. Specific capacity against potential graph of LFP in a Half cell with PEU1 with SBR in ratio 50:50 as the binder at 10 mAg^{-1} current density (0.06C current rate), cycled 50 times between a voltage window 2.5V to 4.2V.

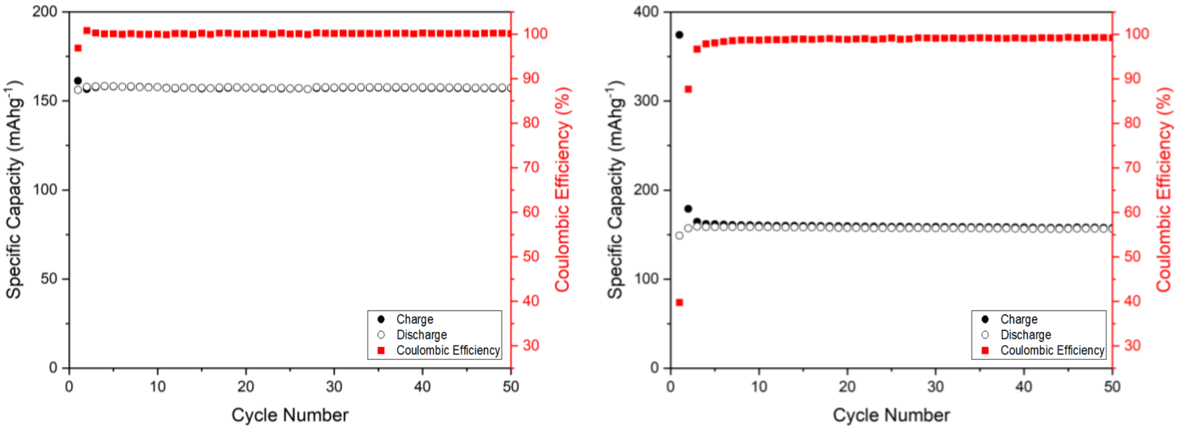

Figure S54. Specific capacity and coulombic efficiency of LFP in a Half cell with PEU1 with SBR in ratio 50:50 as the binder at 10 mAg^{-1} current density (0.06C current rate), cycled 50 times between a voltage window 2.5V to 4.2V.

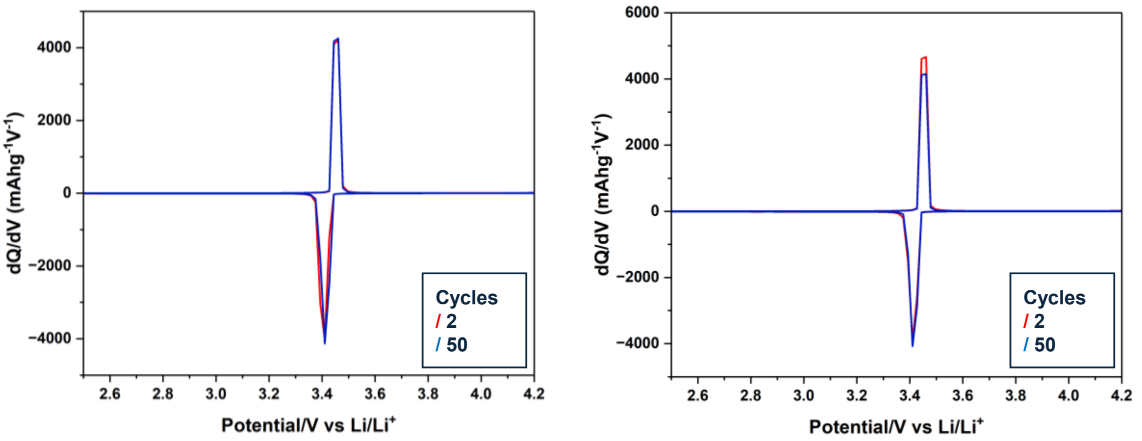

Figure S55. dQ/dV plot of LFP in a Half cell with PEU1 with SBR in ratio 50:50 as the binder at 10 mAg^{-1} current density (0.06C current rate), cycled 50 times between a voltage window 2.5V to 4.2V.

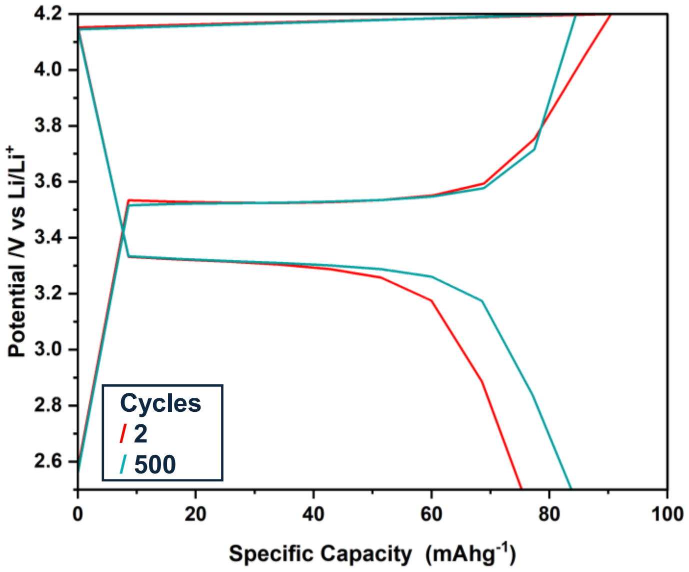

Figure S56. Specific capacity against potential graph of LFP in a half cell with PEU1 with SBR binder in ratio 50:50 at 3C current rate, cycled 500 times between a voltage window 2.5V to 4.2V.

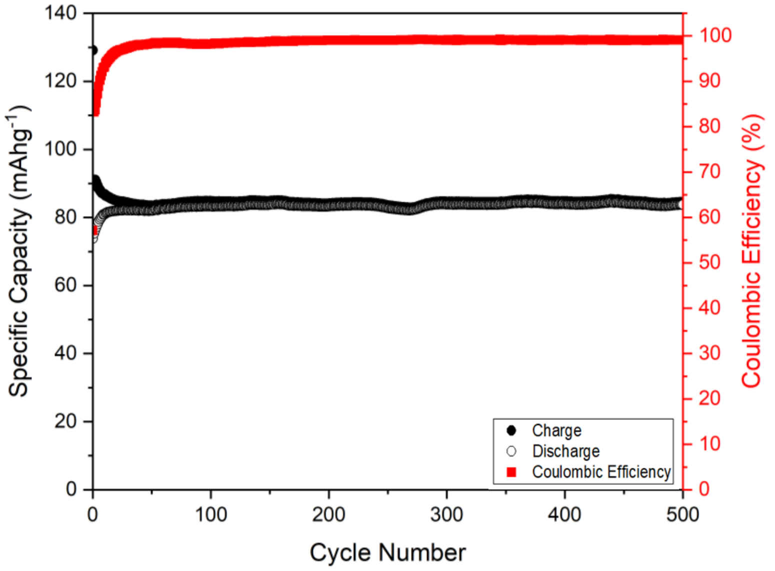

Figure S57. Specific capacity and coulombic efficiency of LFP in a half cell with PEU1 with SBR binder in a ratio 50:50 at 3C current rate, cycled 500 times between a voltage window of 2.5V to 4.2V.

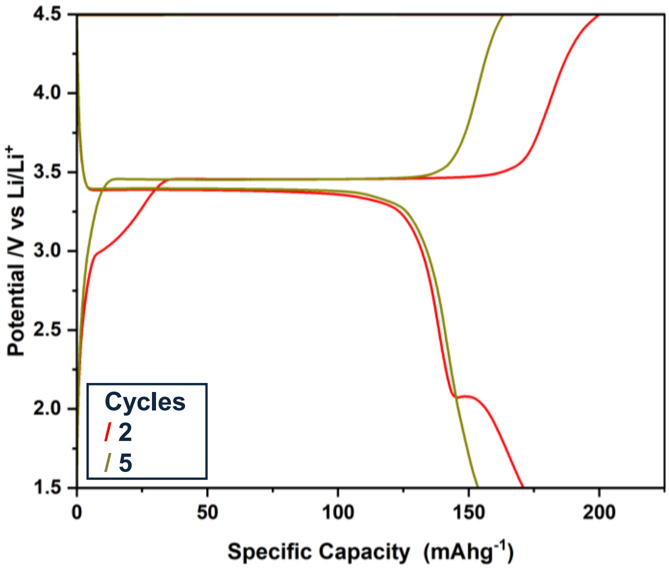

Figure S58. Specific capacity against potential graph of LFP in a half-cell with PEU1 with SBR binder in a ratio 50:50 at 10 mAg^{-1} current density (0.06C current rate), cycled 5 times between a voltage window 1.5V to 4.5V.

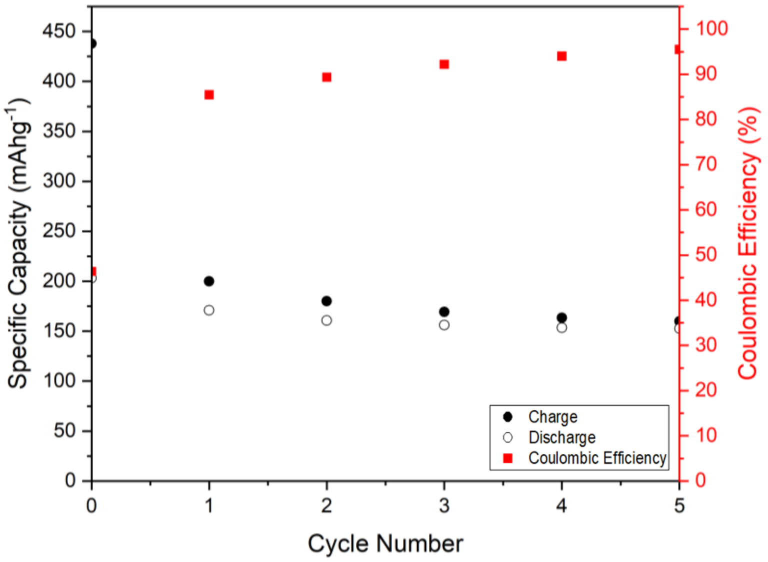

Figure S59. Specific capacity and coulombic efficiency of LFP in a Half cell with PEU1 with SBR in ratio 50:50 as the binder at 10 mAg^{-1} current density (0.06C current rate), cycled 5 times between a voltage window 1.5V to 4.5V.

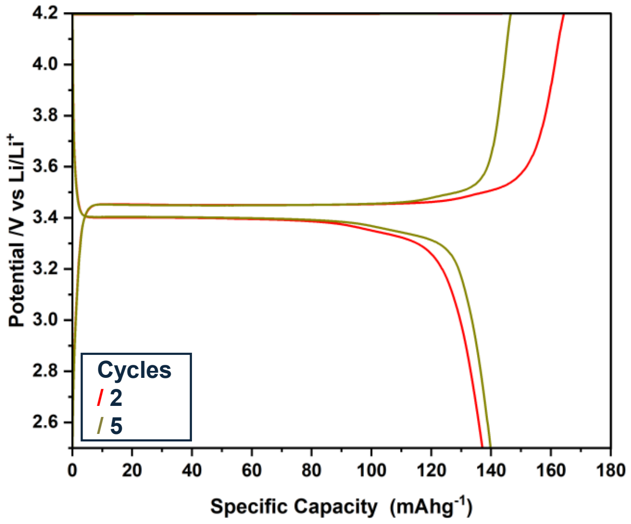

Figure S60. Specific capacity against potential graph of LFP in a half cell with PEU1 with SBR binder in ratio 30:70 at 10 mAg^{-1} current density (0.06C current rate), cycled 5 times between a voltage window 2.5V to 4.2V.

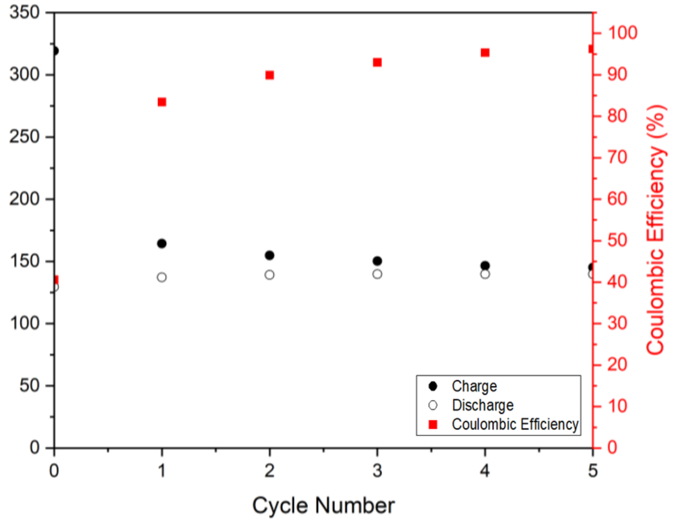

Figure S61. Specific capacity and coulombic efficiency of LFP in a half cell with PEU1 with SBR binder in ratio 30:70 at 10 mAg^{-1} current density (0.06C current rate), cycled 5 times between a voltage window 2.5V to 4.2V.

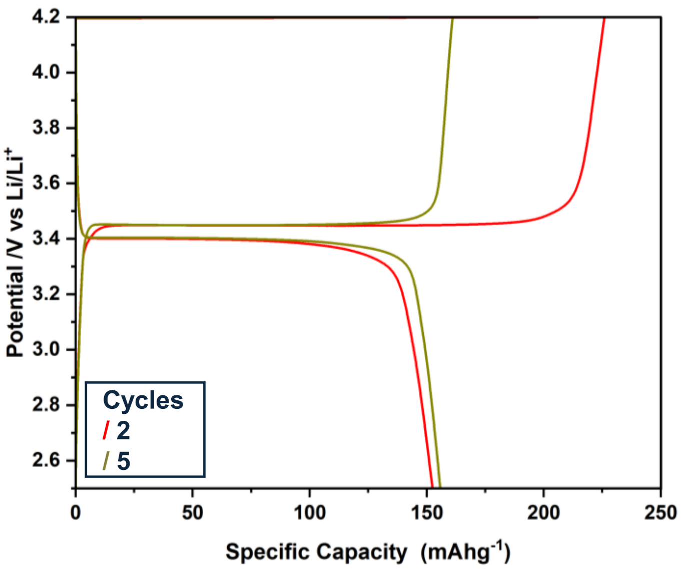

Figure S62. Specific capacity against potential graph of LFP in a half cell with PEU1 with SBR binder in ratio 70:30 as the binder at 10 mAg^{-1} current density (0.06C current rate), cycled 5 times between a voltage window 2.5V to 4.2V.

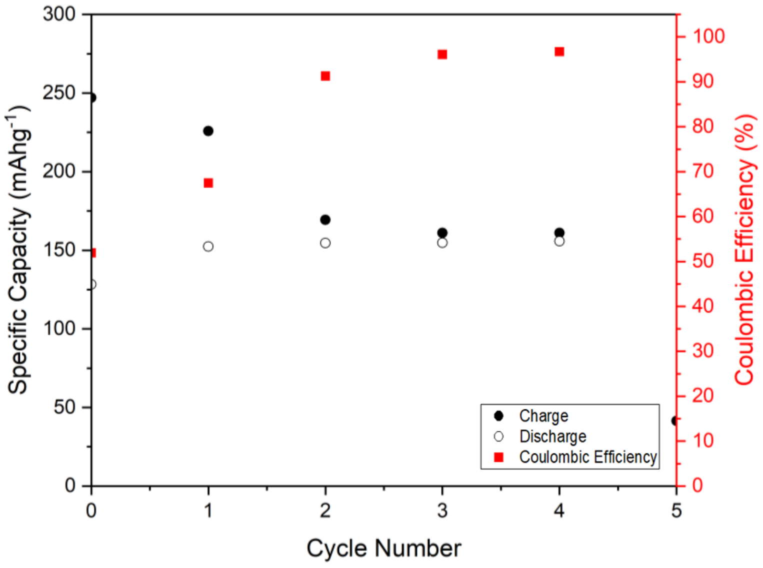

Figure S63. Specific capacity and coulombic efficiency of LFP in a half cell with PEU1 with SBR binder in ratio 70:30 at 10 mAg^{-1} current density (0.06C current rate), cycled 5 times between a voltage window 2.5V to 4.2V.

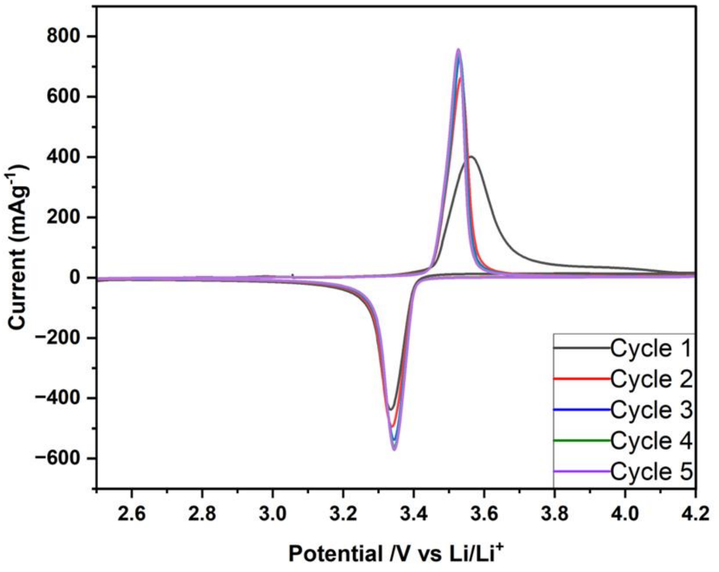

Figure S64. Cyclic voltammogram curves of Half-cell in case of PEU1 with SBR in ratio 50:50 binder, cycled at 0.1 mVs^{-1} for 5 times between a voltage window 2.5V to 4.2V.

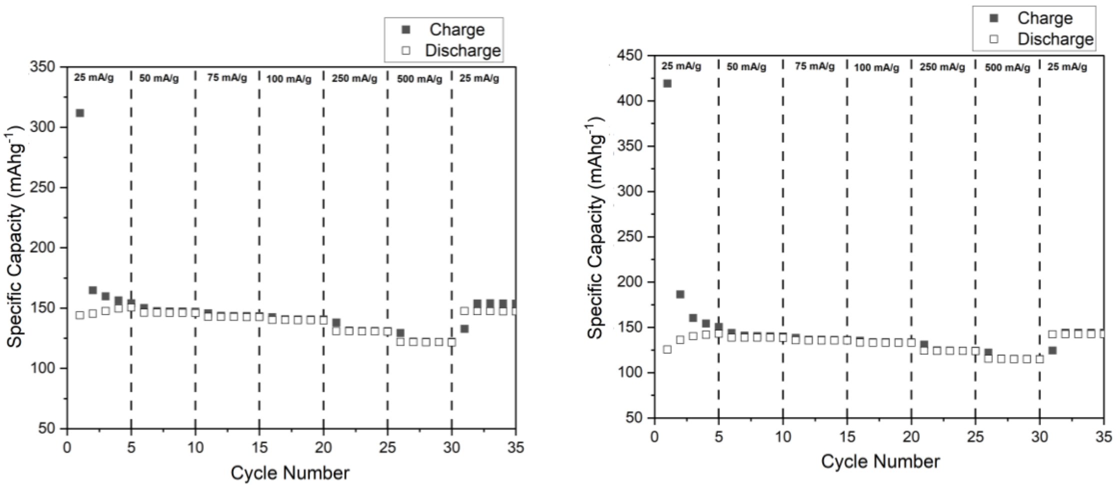

Figure S65. Rate performance of LFP in a Half cell with PEU1 with SBR as the binder in ratio 50:50 at current rate ranging from 25 mAg^{-1} to 500 mAg^{-1}, cycled 5 times at each current density, between a voltage window 2.5V to 4.2V.

7.7. PEU2

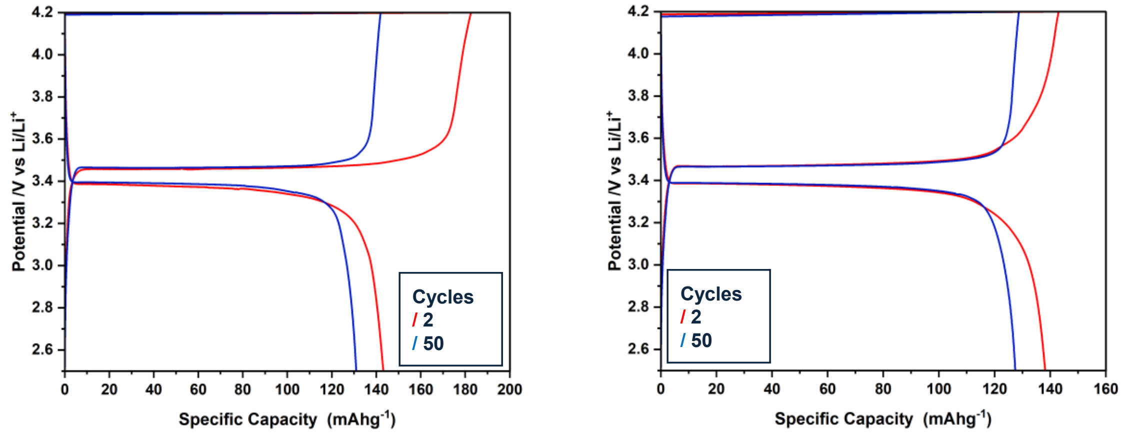

Figure S66. Specific capacity against potential graph of LFP in a Half cell with PEU2 as the binder with a 10 mAg^{-1} current density (0.06C current rate), cycled 50 times between a voltage window 2.5V to 4.2V.

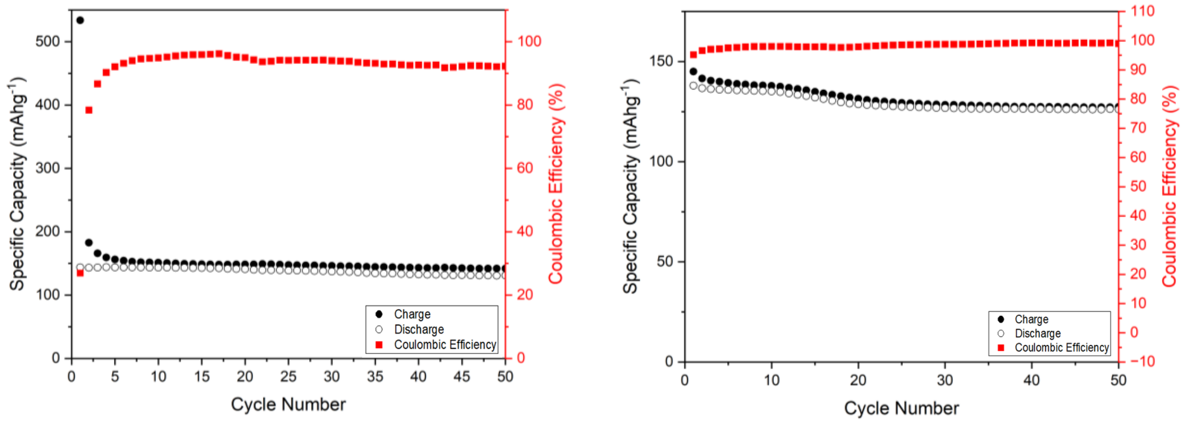

Figure S67. Specific capacity and coulombic efficiency of LFP in a Half cell with PEU2 as the binder at 10 mAg^{-1} current density (0.06C current rate), cycled 50 times between a voltage window 2.5V to 4.2V.

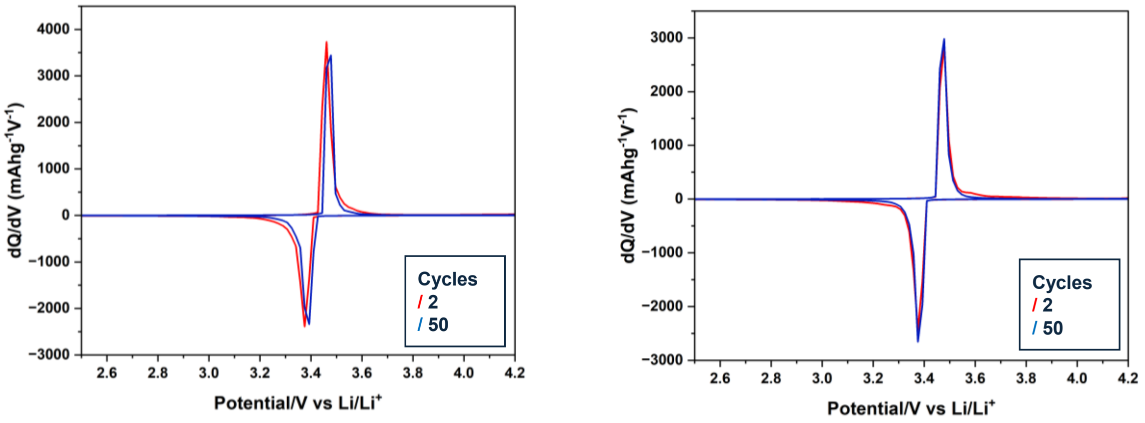

Figure S68. dQ/dV plot of LFP in a Half cell with PEU2 as the binder at 10 mAg^{-1} current density (0.06C current rate), cycled 50 times between a voltage window 2.5V to 4.2V.

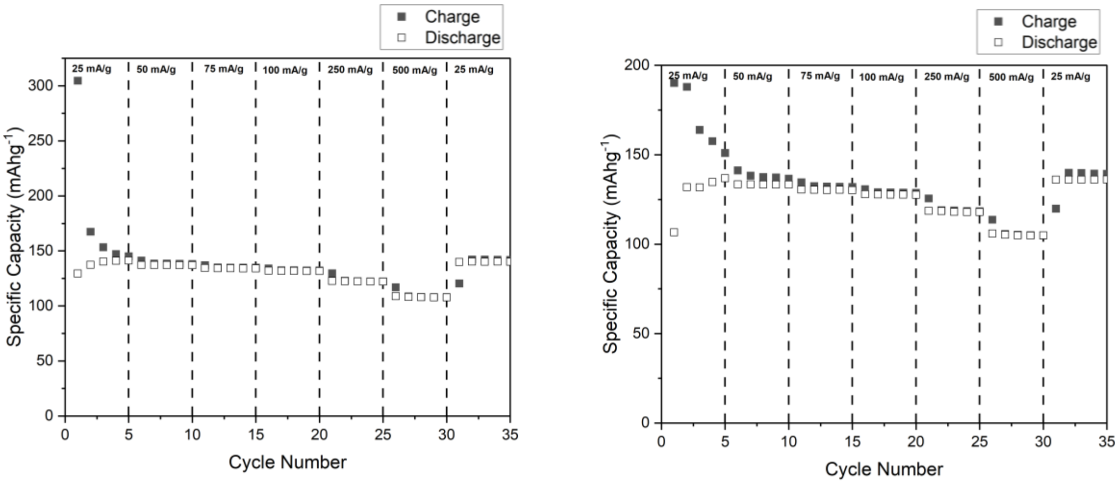

Figure S69. Rate performance of LFP in a Half cell with PEU2 as the binder at current rate ranging from 25 mAg^{-1} to 500 mAg^{-1}, cycled 5 times at each current density, between a voltage window 2.5V to 4.2V.

8. SEM images of electrodes before and after cycling

CMC-Pristine

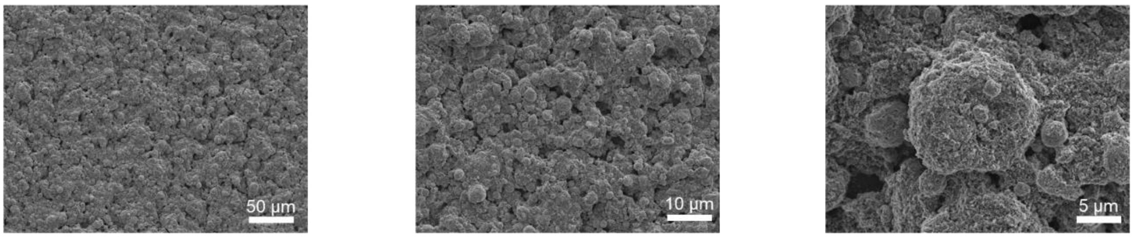

CMC- after 10 Cycles

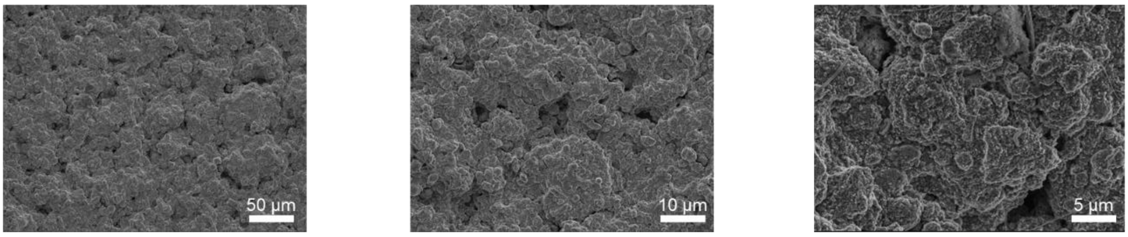

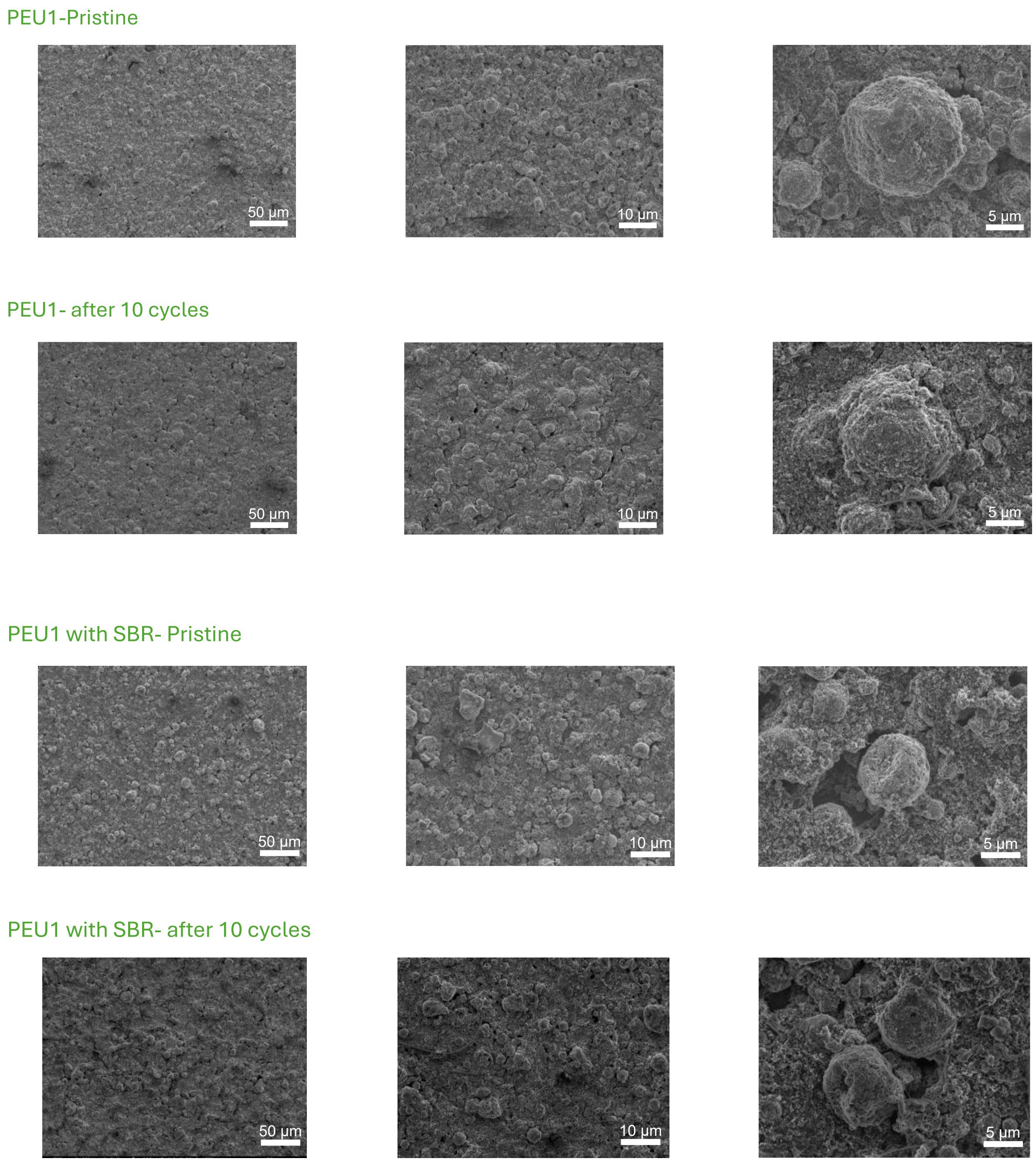

Figure S70. SEM images showing effect on binders CMC, PEU1 and PEU1 with SBR before and after running cells for 10 cycles at 10 mAg^{-1} current density (0.06C current rate).

9. ATR-FTIR images of electrodes before and after cycling

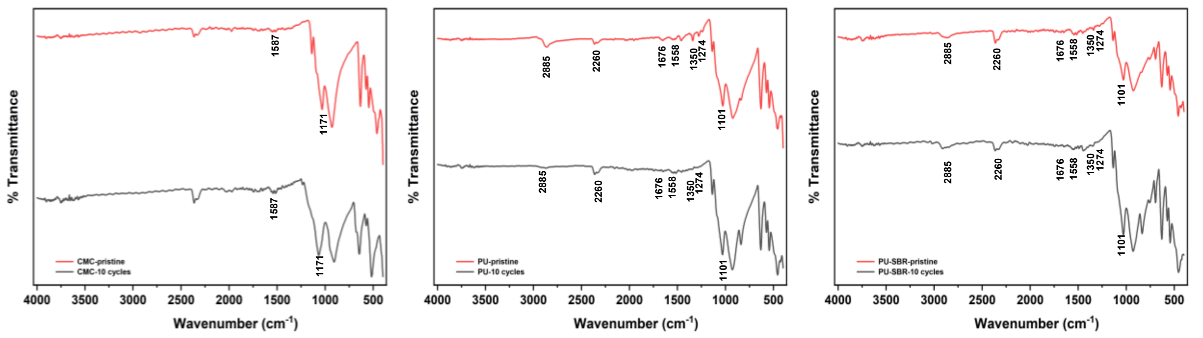

(a) CMC (b) PEU1 (c) PEU1 +SBR

Figure S71. ATR-FT-IR spectra showing effect on binders CMC (a), PEU1 (b) and PEU1 with SBR (c) before and after running cells for 10 cycles at 10 mAg^{-1} current density (0.06C current rate).

10. Impedance data

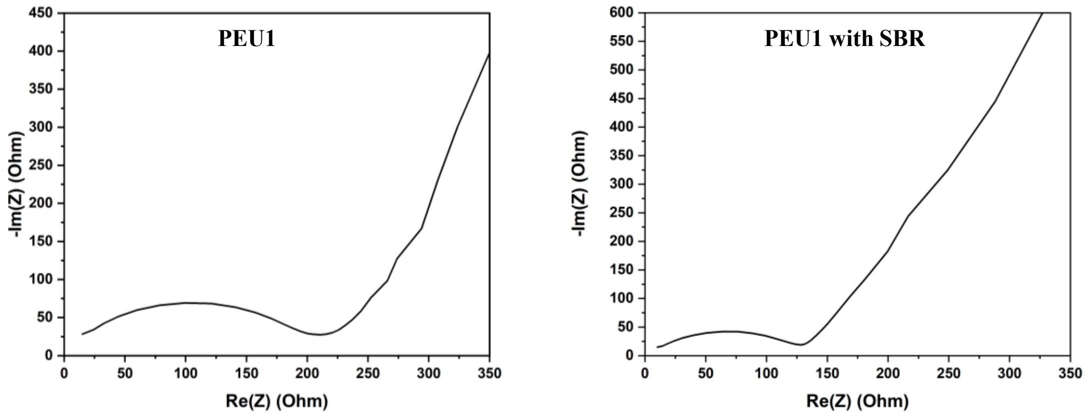

Figure S72. Impedance data showing the effect on binders PEU1 (left) and PEU1 with SBR at 25 mAg^{-1} current density (0.15C current rate).

11. Mechanical properties of binders

<u>Sample preparation and tensile testing</u>

The polymer was dissolved in deionised (DI) water to create a solution with a concentration of 1 g per 5 ml of water. The solution was maintained at a temperature of 40°C to ensure complete dissolution of the polymer. Once fully dissolved, the solution was carefully poured into a Teflon mould to cast a sample with dimensions of 15 mm in width, 65 mm in length, and approximately 2 mm in thickness, suitable for tensile testing.

Before testing, the exact thickness of the specimen was measured and recorded to allow precise stress calculations. To facilitate optical strain measurement during the tensile test, the sample was marked with thin black lines, spaced evenly along its length. These marks served as reference points for tracking strain.

The sample was then subjected to a tensile load at a controlled extension rate of 2 mm/s. During the test, force and extension data were continuously recorded. Following the test, the stress and strain values were calculated and plotted, as shown in Figure 6, to evaluate the material's mechanical properties.

Nanoindentation

Nanoindentation measurements were performed using a Nanoindenter Optics11 Chiaro instrument using the "Displacement Control" modality of the system. A spherical indenter with a diameter of 18 µm and a stiffness of 39.53 N/m was utilized, and indentations were performed using a constant approach velocity of 5 µm/s. Force-displacement curves were obtained and fitted with a Hertz fitting to derive elasticity via the Young's modulus which was modelled using the Optics11Life Data Viewer analysis software. Each sample was mapped in several areas to gather data from different regions of the samples for good representation. Prior to the measurements, the equipment was calibrated against a plastic petri dish.

Nanoindentation analysis was performed on all three polymers using the Oliver–Pharr method to characterize their mechanical properties. The analysis began by determining the stiffness (S) from the unloading segment of the load (P)–displacement (h) curves. A power-law fit was applied to this segment, using parameters B, hf, and m where hf is the residual indentation depth, and B and m are fitting constants derived from the curve.

The contact depth was then calculated, accounting for a geometric constant ε associated with the spherical indenter tip used in this experiment. This constant accounts for the geometry of the indenter in estimating the contact area accurately. The contact area was evaluated based on the calculated contact depth.

Hardness (H) was determined by dividing the maximum applied load by the contact area. The reduced modulus (Er) was calculated using the stiffness (S) and the contact depth multiplied by the constant specific to the indenter geometry and material properties. These calculations provided insights into the hardness and reduced modulus of each polymer, as characterised by the Oliver–Pharr method.

Peel test – method

A Steel plate of 70mm x 40mm x 2mm was used for 180-degree peel test. An electrode sample of 60mm x 25mm was carefully stuck foil side down onto double sided tape. A length of double-sided tape with a tail was then carefully applied to the exposed electrode material – a steel cylindrical roller was then rolled several times to ensure good adhesion. The top of the tape was clamped into the upper jaws. A McMesin tensile machine was used at speed set to 50mm/min. Force vs Displacement data was recorded.

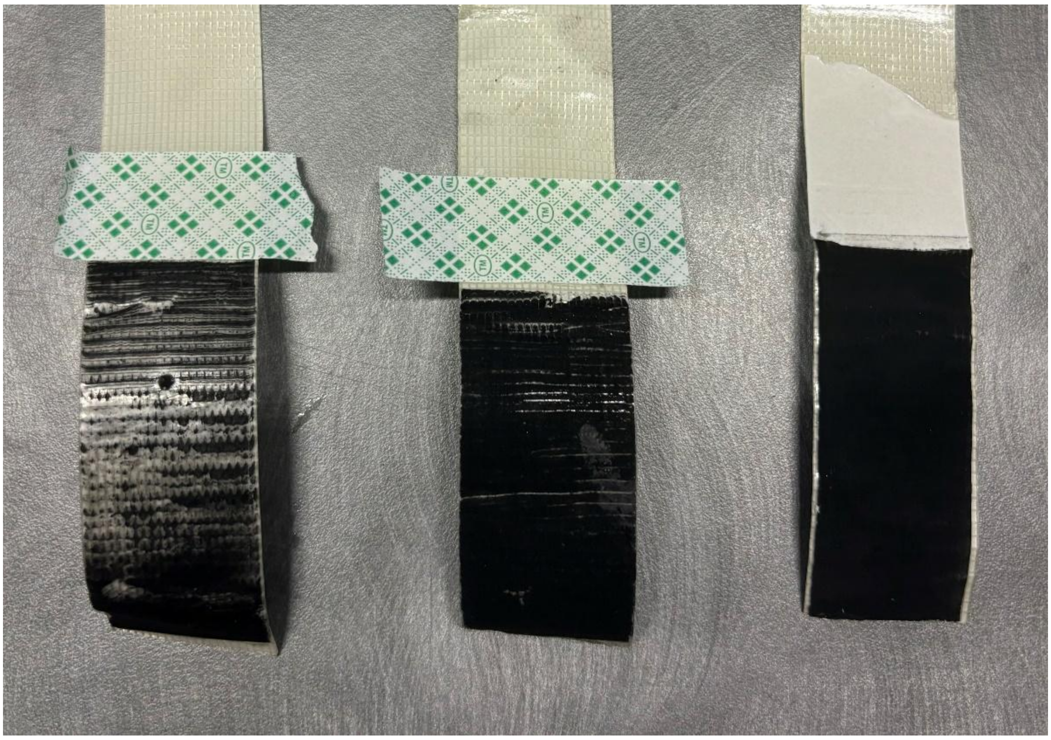

Figure S73. Samples from the peel test. From left to right: PEU1, PEU1+SBR, CMC.

12. Table S2: Comparison of properties/performance of PEU1/PEU1+SBR binder against those of CMC binder.

**Property/Performance**	**PEU1 or PEU1+SBR**	**CMC or CMC+SBR**
Initial Coulombic efficiency (ICE)*	69.6% for PEU1, 96.9% for PEU1+SBR	33.7% for CMC, 27.9% for CMC+SBR
Coulombic efficiency *	99.2% for PEU1, 99.9% for PEU1+SBR	95.3% for CMC, 98.0% for CMC+SBR
Polarisation from dQ/dV study*	50 mV for PEU1, 30 mV for PEU1+SBR	70 mV for CMC, 70mV for CMC+SBR
Specific Discharge Capacity*	148 $mAhg^{-1}$ for PEU1, 157 $mAhg^{-1}$ for PEU1+SBR	142 $mAhg^{-1}$ for CMC, 153 $mAhg^{-1}$ for CMC+SBR
Thermal decomposition	T_{donset}= 238°C, $T_{d,\ 5\%=}$ 330°C, $T_{d,\ 90\%=}$ 385°C for PEU1	295 °C[1]
Electrochemical stability (CV)	Reversible redox behaviour (ΔV = 200 mV for PEU1 and 180 mV for PEU1+SBR)	ΔV = 170 mV
Reduced elastic modulus	95 MPA (PEU1)	375 MPa
Rockwell hardness number	20 (PEU1)	93

Yield strength	7.6 MPa (PEU1)	18 MPa
Adhesion force	19.2 N/cm for PEU1, 18.5 N/cm for PEU1+SBR	0.7 N/cm

at 10 mA g^{-1} current density, cycled over the voltage window 2.5V to 4.2V.

13. Table S3: Comparison of our results with the other aqueous binders reported in the literature

Binder	Specific Discharge capacity (mAhg-1)	CE%	Current rate
CMC (reported herein)	142	95.3	0.06C
CMC+SBR (reported herein)	153	98.0	0.06C
PEGDiamine (reported herein)	150	98.0	0.06C
PEGDiamine+SBR (reported herein)	158	98.2	0.06C
PEU1 (reported herein)	148	99.2	0.06C
PEU1+SBR (reported herein)	157	99.9	0.06C
PEU2 (reported herein)	143	93.7	0.06C
Polyacrylic acid[2]	133	98.8	0.001C
Guar gum[3]	125	94.8	1C
Chitosan[4]	159.4	98.4	0.1C
PVDF[4]	127.9	85.1	0.1C
Sodium Alignate[5]	149	93.7	0.2C

14. Atom economy

Conventional method

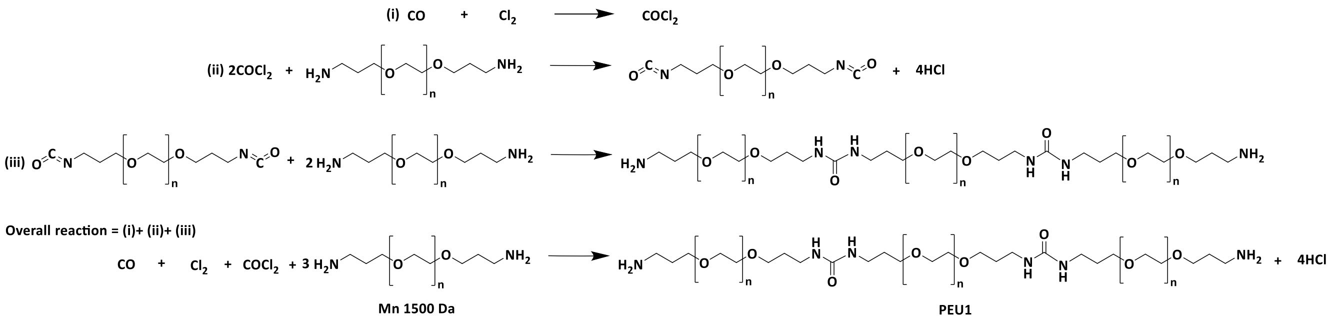

$$\% \text{ Atom economy} = \frac{\text{Molar mass of PEU1}}{\text{Molar mass of (CO + Cl2 + COCl2 + PEG Diamine)}} \text{ X } 100\ \%$$

$$= \frac{4552}{28 + 71 + 99 + 4500} \text{ X } 100\ \%$$

$$= \frac{4552}{4698} \text{ X } 100\ \%$$

$$= \frac{4552}{4698} \text{ X } 100\ \%$$

$$= 96.9\ \%$$

Our method

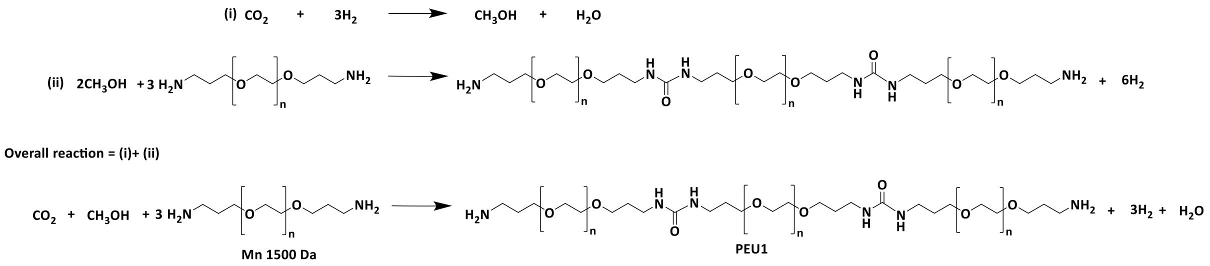

$$\% \text{ Atom economy} = \frac{\text{Molar mass of PEU1}}{\text{Molar mass of (CO2 + CH3OH + PEG Diamine)}} \text{ X } 100\ \%$$

$$= \frac{4552}{44 + 32 + 4500} \text{ X } 100\ \%$$

$$= \frac{4552}{4576} \text{ X } 100\ \%$$

$$= \frac{4552}{4576} \text{ X } 100\ \%$$

$$= 99.5\ \%$$

15. References

1 M. El-Sakhawy, H.-A. S. Tohamy, A. Salama and S. Kamel, *CELLULOSE CHEMISTRY AND TECHNOLOGY Cellulose Chem. Technol*, 2019, **53**, 667–675.

2 Z. P. Cai, Y. Liang, W. S. Li, L. D. Xing and Y. H. Liao, *J Power Sources*, 2009, **189**, 547–551.

3 S. Kaur and S. Santra, *ChemistryOpen*, 2022, **11**, e202100209.

4 K. Prasanna, T. Subburaj, Y. N. Jo, W. J. Lee and C. W. Lee, *ACS Appl Mater Interfaces*, 2015, **7**, 7884–7890.

5 Y. Ding, X. Zhong, C. Yuan, L. Duan, L. Zhang, Z. Wang, C. Wang and F. Shi, *ACS Appl Mater Interfaces*, 2021, **13**, 20681–20688.
